# Beyond single-species models: leveraging multispecies forecasts to navigate the dynamics of ecological predictability

**DOI:** 10.7717/peerj.18929

**Published:** 2025-02-19

**Authors:** Nicholas J. Clark, S. K. Morgan Ernest, Henry Senyondo, Juniper Simonis, Ethan P. White, Glenda M. Yenni, K. A. N. K. Karunarathna

**Affiliations:** 1 School of Veterinary Science, University of Queensland, Gatton, Queensland, Australia; 2 UQ Spatial Epidemiology Laboratory, University of Queensland, Gatton, Queensland, Australia; 3 Wildlife Ecology and Conservation, University of Florida, Gainesville, Florida, United States; 4 DAPPER Stats, Portland, Oregon, United States; 5 Department of Mathematics, Faculty of Science, Eastern University, Chenkalady, Sri Lanka

**Keywords:** Biotic interactions, Community dynamics, Ecological forecasting, Generalized additive model, Stan, State-space model, Vector autoregression

## Abstract

**Background:**

Forecasting the responses of natural populations to environmental change is a key priority in the management of ecological systems. This is challenging because the dynamics of multi-species ecological communities are influenced by many factors. Populations can exhibit complex, nonlinear responses to environmental change, often over multiple temporal lags. In addition, biotic interactions, and other sources of multi-species dependence, are major contributors to patterns of population variation. Theory suggests that near-term ecological forecasts of population abundances can be improved by modelling these dependencies, but empirical support for this idea is lacking.

**Methods:**

We test whether models that learn from multiple species, both to estimate nonlinear environmental effects and temporal interactions, improve ecological forecasts compared to simpler single species models for a semi-arid rodent community. Using dynamic generalized additive models, we analyze time series of monthly captures for nine rodent species over 25 years.

**Results:**

Model comparisons provide strong evidence that multi-species dependencies improve both hindcast and forecast performance, as models that captured these effects gave superior predictions than models that ignored them. We show that changes in abundance for some species can have delayed, nonlinear effects on others, and that lagged, nonlinear effects of temperature and vegetation greenness are key drivers of changes in abundance for this system.

**Conclusions:**

Our findings highlight that multivariate models are useful not only to improve near-term ecological forecasts but also to ask targeted questions about ecological interactions and drivers of change. This study emphasizes the importance of jointly modelling species’ shared responses to the environment and their delayed temporal interactions when teasing apart community dynamics.

## Introduction

Predicting the impacts of environmental change on ecosystem function and biodiversity is a global challenge (Clark et al., 2001; Fredston et al., 2023; IPBES, 2019). Explicit predictions are needed to guide ecological management decisions, inform monitoring programs, and perform scenario planning (Lindenmayer et al., 2012; Tulloch, Hagger & Greenville, 2020). This has led to a growing emphasis on the importance of near-term ecological forecasting to encourage greater reliance on ecological time series data, and on suitable models that can handle the complexities of these data, to generate quantitative forecasts that can be harnessed to guide management decisions (Dietze et al., 2018; Karunarathna, Wells & Clark, 2024; Lewis et al., 2023). The applications of ecological forecasting are broad, including the prediction of soil microbiome compositions (Averill et al., 2021), carbon cycle dynamics (Dietze et al., 2014) and species’ population dynamics (Johnson-Bice et al., 2021; Ward et al., 2014; White et al., 2019).

Forecasts for species population dynamics are especially crucial for conservation planning, stock assessments and other ecological management priorities. However, these forecasts typically focus on only a single species at a time (Lewis et al., 2022; Quinn, 2003; Simonis, White & Ernest, 2021) or on aggregate measures such as species richness, biomass or diversity (Algar et al., 2009; Clark, Kerry & Fraser, 2020; Tonkin et al., 2017). However, key applications of population dynamics forecasts, including changes in ecosystem function and biodiversity loss, are rarely single-species issues (Greenville et al., 2016; Lindenmayer et al., 2012). In addition, because species differ in their niche requirements, ecosystems containing multiple species of interest may require managers to balance competing needs not only between human and ecosystem requirements, but also among different species (*e.g*., Romañach et al., 2022). Finally, species population dynamics are known to be related to one another due to both direct interactions between species (*e.g*., competition) and because species respond to shared environmental drivers (Ovaskainen et al., 2017; Volterra, 1928; Warton et al., 2015). These associations between the dynamics of different species has resulted in extensive research into multivariate population dynamics models, where time series of multiple response variables (such as counts of multiple species or of different age classes for the same species) are jointly modelled (Bunin, 2017; Ives et al., 2003; Paniw et al., 2023; Ward et al., 2010; Ward, Marshall & Scheuerell, 2022). Leveraging the multi-response associations that these models are capable of learning could potentially result in more accurate forecasts and better-informed scenario planning, including approaches to predicting the impacts of species extinctions or the potential spread of invasive species (Ibáñez et al., 2009).

However, despite the potential advantages of multi-species dynamic models, their implementation is still rare in ecological applications in general and in population forecasting specifically. A recent review of 178 near-term ecological forecast applications, with targets ranging from wildlife population trajectories to fisheries stocks and algal bloom forecasting, found that only 10 (5.6%) used multivariate models to generate and evaluate forecasts (Lewis et al., 2022). This finding is in line with an earlier review of population dynamics models for informing marine reserve design, which found that only 1 of 34 studies considered multi-species dynamics (Gerber et al., 2003).

The rarity of multi-species population dynamic forecasting is likely due in part to the higher computational costs and statistical complexity needed to formulate multivariate population dynamic models that incorporate real world complexities in ecological data (Karp et al., 2023). Forecasting the abundances of multiple species is particularly difficult, for several reasons. Many biological and physiological processes influence population dynamics (Hampton et al., 2013; Quinn, 2003), and species often exhibit complex responses to external drivers (including non-linear responses and lags; Cárdenas et al., 2021; Karunarathna, Wells & Clark, 2024). Moreover, temporal autocorrelation is often prevalent in abundance time series data (due to population processes; Ives, Abbott & Ziebarth, 2010), which can be difficult to address in ecological models. Finally, because monitoring wildlife is challenging, data complexities (*e.g*., irregular sampling intervals, observation errors, missing samples, and outcomes manifesting as discrete counts with meaningful lower and/or upper bounds) bring additional challenges into an already complicated modelling environment (Clark & Wells, 2023). In combination, these issues often make population time-series data unsuitable for traditional modelling approaches such as regression or simple time series models. Managers may also have differing needs for forecasts, ranging from predicting the most accurate near-term population sizes to exploring potential responses to differing management scenarios (Clark et al., 2001; Lewis et al., 2023; Lindenmayer et al., 2012; Moustahfid et al., 2021).

One area of ecological modelling that has embraced multi-species approaches is joint species distribution models (JSDMs), which leverage similarities in species’ spatial patterns to predict the distributions of multiple species in space and time (Clark et al., 2016; Norberg et al., 2019; Powell-Romero et al., 2023; Thorson et al., 2016; Tobler et al., 2019). Many of these models only consider spatial data, but some recent advances have included time-series structures that can learn multi-species dependencies (Abrego et al., 2021; Ovaskainen et al., 2017; Ruiz-Moreno, Emslie & Connolly, 2024). While forecasting multispecies population dynamics remains challenging, these types of models have the potential to provide valuable insights for forecast applications. Theory and experimental evidence support the idea that learning from multiple species should improve population forecasts. For example, a recent experimental study induced changes in the abundance of competitors, resulting in altered species interactions that impacted the accuracy of single-species forecasts (Dumandan et al., 2024). Other work has shown that incorporating information from other species—either by including lagged observations of other species as predictors in single-species models (Abrego et al., 2021; Daugaard et al., 2022) or by building temporal JSDMs with multi-species autoregressive terms (Hampton et al., 2013; Mutshinda, O’Hara & Woiwod, 2009; Ovaskainen et al., 2017; Ruiz-Moreno, Emslie & Connolly, 2024)—improves the accuracy of ecological predictions. But despite these findings, the broader use of multi-species forecasts as an ecological application remains unexplored. Validation of multi-species forecasts, and comparisons against forecasts from simpler single species models, have generally been limited to either in-sample predictive measures (Ruiz-Moreno, Emslie & Connolly, 2024; Sandal et al., 2022) or one-step ahead correlation measures (Abrego et al., 2021; Ovaskainen et al., 2017). We are not aware of any studies that compare single species *vs*. multi-species forecasts beyond a single time step. This is problematic because most forecast applications typically require predicting multiple time steps into the future to assess near-term management needs or responses to likely future scenarios (*i.e*., loss of important species, shifts in important drivers). Moreover, most multi-species time series models fail to incorporate one or more of the many important real-world complexities—observation errors, missing values, non-linear responses to environmental drivers, and latent temporal dynamics—that plague real-world forecasting applications (Clark & Wells, 2023; Daugaard et al., 2022; Holmes, Ward & Scheuerell, 2014; Royle & Nichols, 2003). This combination of a limited exploration of the utility of multi-species models for ecological time series applications and the need to incorporate more complex modeling structures constitutes a major gap in our ability to tackle realistic forecasting applications.

Here we evaluate whether models that incorporate multi-species relationships can improve near-term population forecasts using data from a long-term ecological monitoring study where there is evidence of both direct biotic interactions between species (Bledsoe & Ernest, 2019; Christensen, Simpson & Ernest, 2019; Ernest & Brown, 2001; Heske, Brown & Mistry, 1994; Lima et al., 2008) and shared responses to environmental factors (Christensen, Harris & Ernest, 2018). Using the framework of dynamic generalized additive models developed by Clark & Wells (2023), we build a series of models that learn species’ shared environmental responses and temporal dependencies to make inference about environmental and biotic factors that relate to community dynamics. Our models highlight how several key challenges can be tackled when modelling the dynamics of multiple species, including how to estimate environmental effects that change nonlinearly over increasing lags, how to capture unobserved temporal autocorrelation, and how to estimate lagged temporal dependencies among species. We then test whether the incorporation of these biotic dependence structures improves forecasts compared to simpler single-species models over multiple near-term timescales (up to 12 months) using penalized in-sample performance criteria and out-of-sample forecast metrics. Finally, we demonstrate how these models can be used to perform perturbation experiments for assessing community responses to shifts in key species abundances and to changes in environmental drivers with shared species responses. Because these multi-species dynamic models integrate both species interactions and complex environmental dependencies, our study shows that they can provide a deeper understanding of ecological dynamics while generating more accurate forecasts and predictions for scenario planning. These models are broadly applicable to time-series data, providing a versatile tool for conducting time-series based forecasting to meet the wide-ranging needs of both basic and applied research. Note that portions of this text were previously published as part of a preprint (https://doi.org/10.32942/X2TS34).

## Materials and Methods

We first describe the study system to outline why it is suitable for testing whether multi-species models lead to better ecological forecasts compared to single-species models. Second, we describe our full dynamic model, from which we can make inferences about the processes that drive community dynamics. Third, we describe how we compare this model to simpler models in an iterative forecasting exercise to ask whether models that include multi-species dependencies (a) improve in-sample fits to the observed data and (b) provide better out-of-sample near-term predictions.

### Rodent capture data

Our data come from the Portal Project, a long-term monitoring study of a desert rodent community (Brown, 1998; Ernest et al., 2020) that has been undergoing active forecasting since 2016 (White et al., 2019). The Portal Project is based in the Chihuahuan Desert near Portal, Arizona. The sampling design includes 24 experimental plots (50 m × 50 m), each containing a grid of 49 baited traps (Brown, 1998; Ernest et al., 2020). The design uses three experimental treatments. In control plots (*N* = 10), holes in the fence are large enough to allow free access for all rodents. Full rodent removal plots (*N* = 6) have fences with no holes. Kangaroo rat exclosures (*N* = 8) have fences with holes to allow passage of all rodents except kangaroo rats (*Dipodomys* genus). Investigators close holes during trapping to ensure all captured rodents are residents. Trapping follows the lunar monthly cycle, but weather and other disruptions result in missing observations (~5% on average; Dumandan, Yenni & Ernest, 2023).

The Portal dataset exhibits many of the complexities that confront population forecasting. These include observation errors due to imperfect detection, missing samples due to weather or other issues (*e.g*., global pandemics), and discrete counts of captured individuals for many species (20 rodent species) that include large numbers of zeros, multi-species dependencies and upper bounds set by the number of traps (Ernest et al., 2020). Environmental drivers, including temperature and measures of primary production, exhibit lagged and nonlinear impacts on rodent breeding, activity rates, and population dynamics (Brown & Ernest, 2002). Moreover, the rodent species at Portal are known to compete for resources in complex ways, and these biotic interactions are postulated to have important consequences for variation in population dynamics. In other words, the Portal Project exhibits many of the complexities that make the ecological forecasting of species populations particularly difficult, making it an ideal real-world test case for exploring whether multi-species models can provide better near-term predictions than single species models.

**Figure 1:**
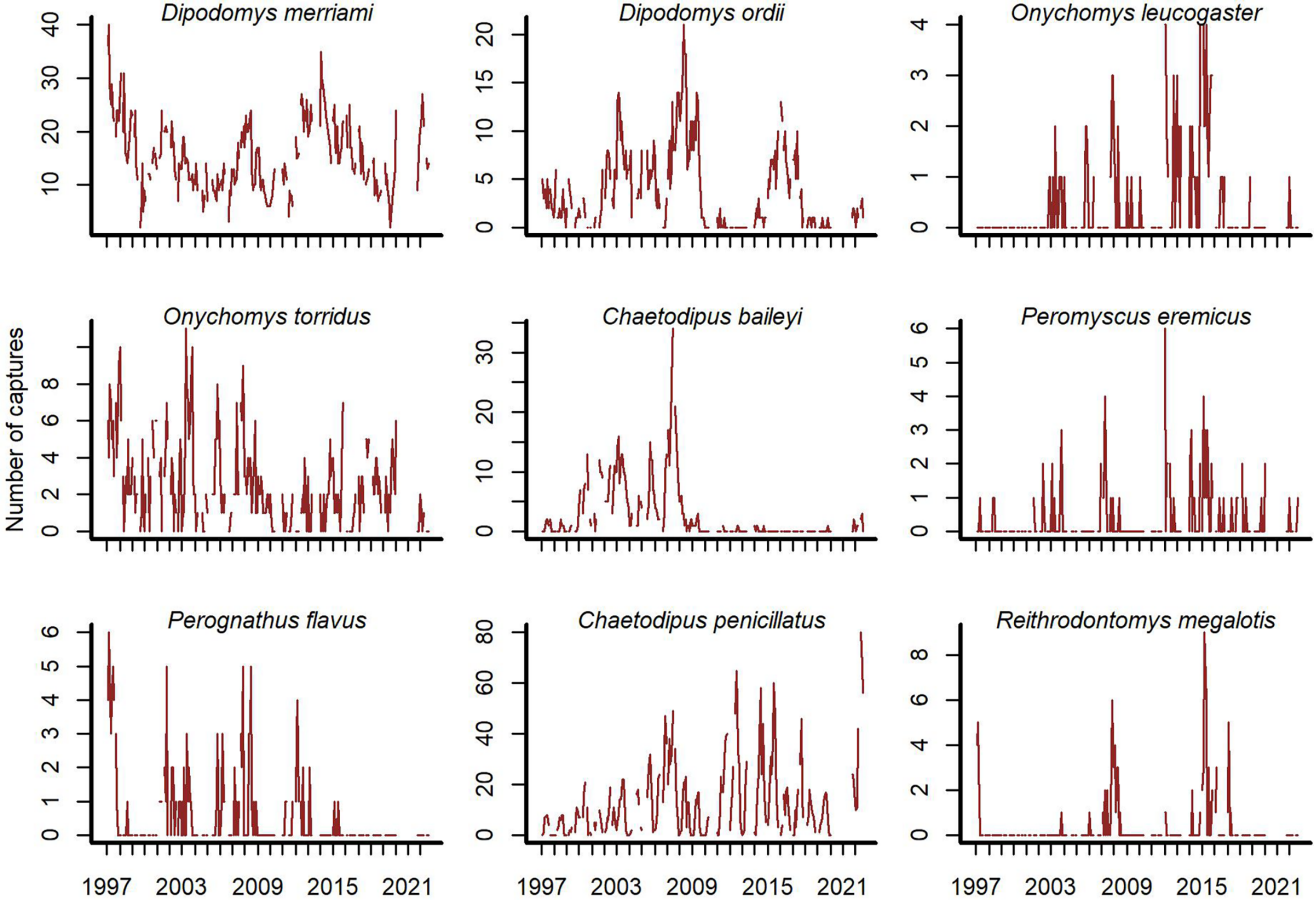
Rodent capture data from the Portal Project for the period December 1996 to August 2022. Counts are total captures across long-term control plots. Blanks are missing values.

Open-source software exists to access the Portal Project data (Christensen et al., 2019; Simonis et al., 2022). We used the *portalr* R package to extract trapping records from the Portal data (version 3.134.0; downloaded October 2022; https://doi.org/10.5281/zenodo.7255488). Our study focused on rodent captures from the long-term control plots for the period December 1996–August 2022. The data has records for 20 rodent species, but some are rarely captured. We excluded species if they were observed in <10% of trapping sessions. We did this to focus inferences on species with the most influence on community dynamics. Each temporal observation was a vector of total captures on long-term control plots for the nine remaining species at a given sampling time (Fig. 1).

### Covariate measurements

Rodent populations at Portal, and the associated number of captures recorded during sampling, depend on environmental conditions that reflect resource availability and seasonal breeding signals. We therefore modelled species’ responses to environmental variation using the Normalized Difference Vegetation Index (NDVI, representing a signal of resource availability) and minimum temperature (which strongly reflects variation in seasonal breeding behaviours; Dumandan et al., 2024) as covariates. Hourly air temperature (°C) is recorded by an automated weather station on site, which we used to calculate a daily minimum, while Landsat images are used to calculate NDVI for an area 1,000 m in radius around the geographical centre of the Portal Project site (accessed on a 2-week basis from the US Geological Service Earth Resources Observation and Science Center; https://www.usgs.gov/centers/eros). Measurements for both covariates (daily minimum temperature and bimonthly NDVI) were then converted to monthly averages. We extracted covariate data from one year before the start of captures (from January 1995) so we could calculate lagged and moving average versions. See Ernest et al. (2020) for further details of environmental measurements, including their spatial and temporal resolutions.

### Model description

**Figure 2:**
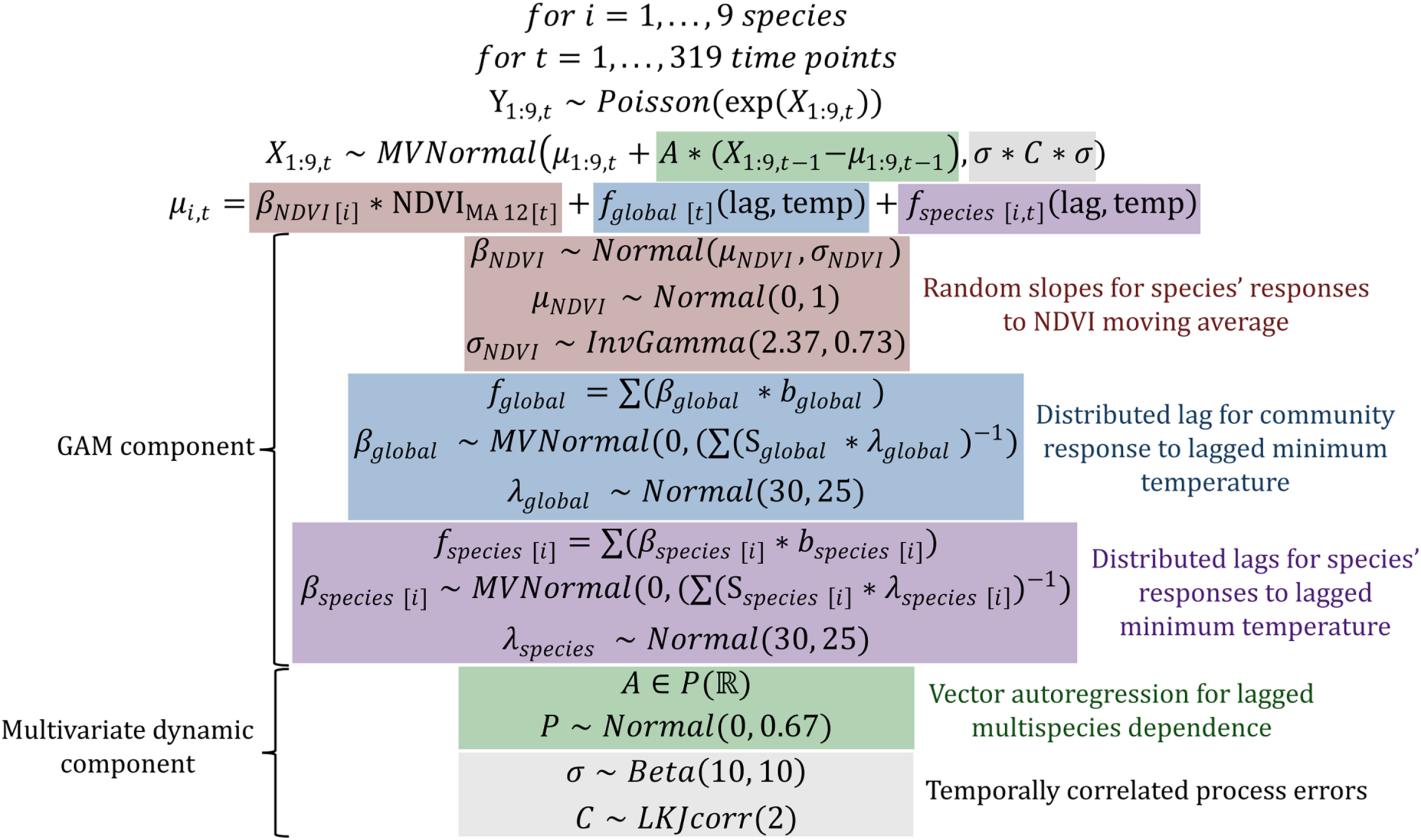
Model definition and prior definitions for the multivariate *GAM-VAR* model. Coloured boxes highlight the five main components of the dynamic latent state model. Here, *Y_1:9,t_* is a vector of observed counts of trapped individuals for the nine rodent study species at time point *t*, which we assume can be modelled as random draws from a Poisson distribution whose logged mean (*X_1:9,t_*, representing the latent State model) follows a multivariate Gaussian distribution. The mean of the multivariate latent State model *X* is decomposed into penalized effects of NDVI and minimum temperature (*µ_1:9,t_*, making up the GAM components whose prior distributions are described in the red, blue and purple boxes) and a first order Vector Autoregression (with a 9 × 9 autoregressive coefficient matrix *A*, whose prior distribution is described in the green box). GAM parameters to be estimated include the *β* regression coefficients, the mean (*µ_NDVI_*) and variance (*σ_NDVI_*) of the hierarchical NDVI_MA 12_ effects, and the *λ* smoothing penalties. The covariance matrix of the latent State model *X* is decomposed into a vector of variance components *σ* (representing unmodelled process errors) and a correlation matrix *C* (representing contemporaneous correlations in process errors), whose prior distributions are described in the grey box. Required data objects for the model are the observations *Y*, the covariate design matrix (including columns for the 12-month moving average of observed NDVI and the values of the evaluated distributed lag effects *b_global_* and *b_species [1:9]_*) and the distributed lag penalty matrices *S_global_* and *S_species [1:9]_*.

There were several aspects of the data we needed to consider when designing our model. Total rodent captures showed both short- and long-term fluctuations (Fig. S1). Captures for individual species also undulated over multi-annual cycles and were positively autocorrelated at lags up to 20 months (Figs. S2 and S3). To test whether multi-species information improves model performance, we needed to model these dynamics using a multivariate dependence structure. Second, we needed to leverage community information to estimate each species’ time-delayed response to variation in vegetation and temperature. Because species’ responses to environmental change in this system are expected to be delayed and nonlinear (Brown & Ernest, 2002), we used splines to model these responses. Rodent captures were modelled as $Poisson$ observations of a latent state model that was composed of a hierarchical generalized additive model (GAM) component (to capture shared, nonlinear environmental responses) and a multivariate dynamic vector autoregressive component to capture multi-species dependence. This State-Space model was designed to explicitly address several of the key challenges that plague population dynamics forecasting by leveraging (1) a discrete observation model to appropriately deal with count-valued time series with many zeros; (2) a latent real-valued state model with a separate error component to deal with imperfect detection; (3) hierarchical effects and penalized splines to model species’ nonlinear environmental responses and (4) a vector autoregression (VAR) component to capture both lagged and contemporaneous dependencies among species’ estimated latent states. The full description for this model, which we abbreviate to *GAM-VAR*, is shown in Fig. 2. The GAM component of the model consisted of hierarchical NDVI and minimum temperature effects. The structural forms of these functions were informed by theory and exploration of covariate time series (shown in Figs. S4, S5). We used a 12-month moving average of NDVI ( $NDV{I_{MA12}}$) because we expected rodent populations to respond gradually to vegetation change, whereby productive years (represented as timepoints with relatively high NDVI values) will likely result in delayed population increases as rodents are able to cache more seeds and make use of the higher availability of shelter spaces (Brown, 1998; Brown & Ernest, 2002). Our model assumed linear effects of $NDV{I_{MA12}}$, equivalent to a hierarchical slopes model. The partial pooling properties of this model allowed us to regularize weakly informed slopes toward a community average. Responses to temperature were estimated using a hierarchical distributed lag model in which nonlinear effects of minimum temperature varied smoothly with increasing lag. These effects were constructed as tensor products of four cubic basis functions for lag and three thin plate basis functions for minimum temperature. To allow our model to capitalize on multi-species learning, we included a shared community-level response ${f_{global}}\left( {Mintemp,lag} \right)$ and species-level deviation responses ${f_{species\left[ i \right]}}\left( {Mintemp,lag} \right)$. The sum of these effects allowed each species to show a different temperature response from the wider community, but only if there was information in the data to support such a deviation. We used lags of up to 6 months in the past because this time window is likely sufficient to capture rodent behavioral and breeding responses to seasonal temperature change (Karunarathna, Wells & Clark, 2024).

A VAR of order 1 captured lagged multi-species dependence, where $A$ was a nonsymmetric 9 × 9 matrix of autoregressive coefficients (Fig. 2). Diagonal entries of $A$ described density-dependence, or the effect of a species’ dynamic process (at time $t$) on its own lagged values (at $t - 1$). Off-diagonals represented cross-dependencies that could provide useful biological insights into interspecific interactions. For example, the entry in $A$**[2,3]** described the effect of species $3$’s dynamic state at time $t - 1$ on the current state estimate for species $2$ (at time $t$). To encourage stability and prevent forecast variance from increasing indefinitely, we enforced stationarity following methods described in Heaps (2023). Briefly, a multistep process was used to map the constrained $A$ matrix to unconstrained partial autocorrelations $P$, which allows for meaningful prior elicitation about the structure of *A* while enhancing computational efficiency. Process errors were allowed to be contemporaneously dependent to capture any unmodelled correlations in species’ latent states. Priors for all model components are shown in Fig. 2 and described in detail in the accompanying R code.

### Evaluating whether multi-species dependencies improve prediction performance

We formally tested whether learning from multiple species improved our model’s predictions using prediction-based model comparisons. To do so, we estimated a series of benchmark models that acted as natural simplifications of the *GAM-VAR* by eliminating multi-species components in a stepwise manner. The first benchmark model used the same hierarchical GAM linear predictor as the *GAM-VAR* but replaced the multi-species VAR(1) dynamics with an AR(1) process. This model (called *GAM-AR* in subsequent sections) eliminated the covariances and temporal cross-dependencies among species’ latent states, allowing us to ask whether the multivariate dynamic component was supported for improving model fit. Next, we further simplified the *GAM-AR* by removing the hierarchical environmental response functions from the linear predictor. This forced the model to learn environmental responses for each species without using information from other species in the data (*GAM-AR no pooling*). The final benchmark, referred to as *AR*, also used independent AR(1) states but removed the GAM component entirely. Because this model only learned from past observations, comparisons against it helped us understand how covariate responses impacted our models’ predictions and inferences. Each candidate model was trained on all observations (through August 2022, *N* = 319 timepoints). Models were then compared using Pareto-smoothed importance sampling leave-one-out cross-validation (PSIS-LOO), a method that reweights posterior draws to estimate leave-one-out pointwise prediction accuracy using estimated log predictive density (ELPD) values (Vehtari, Gelman & Gabry, 2017).

To adequately evaluate competing forecast models, it is also necessary to perform out-of-sample validation (Clark et al., 2022; Harris, Taylor & White, 2018; Lewis et al., 2022). This is particularly important because LOO-CV is designed to ask how models would generalize to new observations *within* the training window. This metric does not evaluate a time series model’s ability to forecast, as information from future timepoints is used to influence predictions for previous time points. To evaluate forecasts in a way that respected the temporal nature of our forecasting exercise, we used exact leave-future-out cross-validation in an iterative expanding window framework. Models were re-trained on the first 273 time points (~22 years), with the subsequent 12 time points (through November 2019; selected to avoid a large sampling gap due to the COVID-19 pandemic) used to evaluate forecasts. This allowed us to gauge how models might perform in a forecast scenario, but it only provided a single comparison. To further scrutinize models, we retrained models on the first 75, 115, 154, 194, and 233 observations, and evaluated the subsequent 12 observations in each cross-validation fold. All forecast comparisons used an evenly weighted combination of two proper multivariate scoring rules. We chose the variogram score, which penalizes distributions that do not adequately capture correlations in test observations, and the energy score, which ignores correlations but penalizes forecasts if they are not well-calibrated (Scheuerer & Hamill, 2015).

### Estimation

We estimated posterior distributions with Hamiltonian Monte Carlo in Stan (Carpenter et al., 2017; Stan Development Team, 2022), specifically the *cmdstanr* interface (Gabry & Češnovar, 2021). Stan’s algorithms provide state-of-the-art diagnostics for probabilistic models (Betancourt, 2017). For example, Hamiltonian Markov chains diverged when attempting to estimate minimum temperature deviations for some species in the *GAM-VAR*. Our data were not informative enough to learn how, or even if, these species responded to temperature change in ways that differed from the community response. Stan’s diagnostics guided us to a model that could be reliably estimated, which included species’ level distributed lag functions for the four most frequently captured species (*D. ordii*, *D*. *merriami*, *Onychomys torridus* and *C. penicillatus*). Posteriors were processed in R 4.3.1 (R Core Team, 2023) with the *mvgam* R package (Clark & Wells, 2023). Traceplots, rank normalized split-R ^ and effective sample sizes interrogated convergence of four parallel chains. Each chain was run for 500 warmup and 1,600 sampling iterations. R code to replicate all analyses and produce figures is included in the Supplemental Materials and is permanently archived on Zenodo (https://doi.org/10.5281/zenodo.14607006).

## Results

### Modeling relationships among species improves prediction performance

Our data included total captures for nine rodent species over 319 time points. All models showed adequate convergence and posterior exploration, and randomized quantile residuals showed no obvious evidence of unmodelled temporal or systematic variation (Figs. S6, S7). However, in-sample performances differed among models, with models that leveraged multi-species information producing higher ELPD scores compared to simpler models (Table 1). This was the case for all stepwise comparisons apart from one: although the *GAM-AR*, which used partial pooling to learn species’ environmental responses, was favoured over the simpler *GAM-AR no pooling*, overlapping ELPD standard errors suggested there was still large uncertainty about the magnitude of this difference (Table 1).

**Table 1:** In-sample (hindcast) validation metrics for competing models. Approximate Pareto-smoothed importance sampling leave-one-out cross-validation (PSIS-LOO) was used to compute the Estimated Log Predictive Density (ELPD) of competing models. A higher ELPD indicates a model is expected to generalize better to new data within the training window.

Model	ELPD difference	SE of ELPD difference
GAM-VAR	0.0	0.0
GAM-AR	−15.5	8.3
GAM-AR no pooling	−22.1	7.0
AR	−74.3	12.6

**Figure 3:**
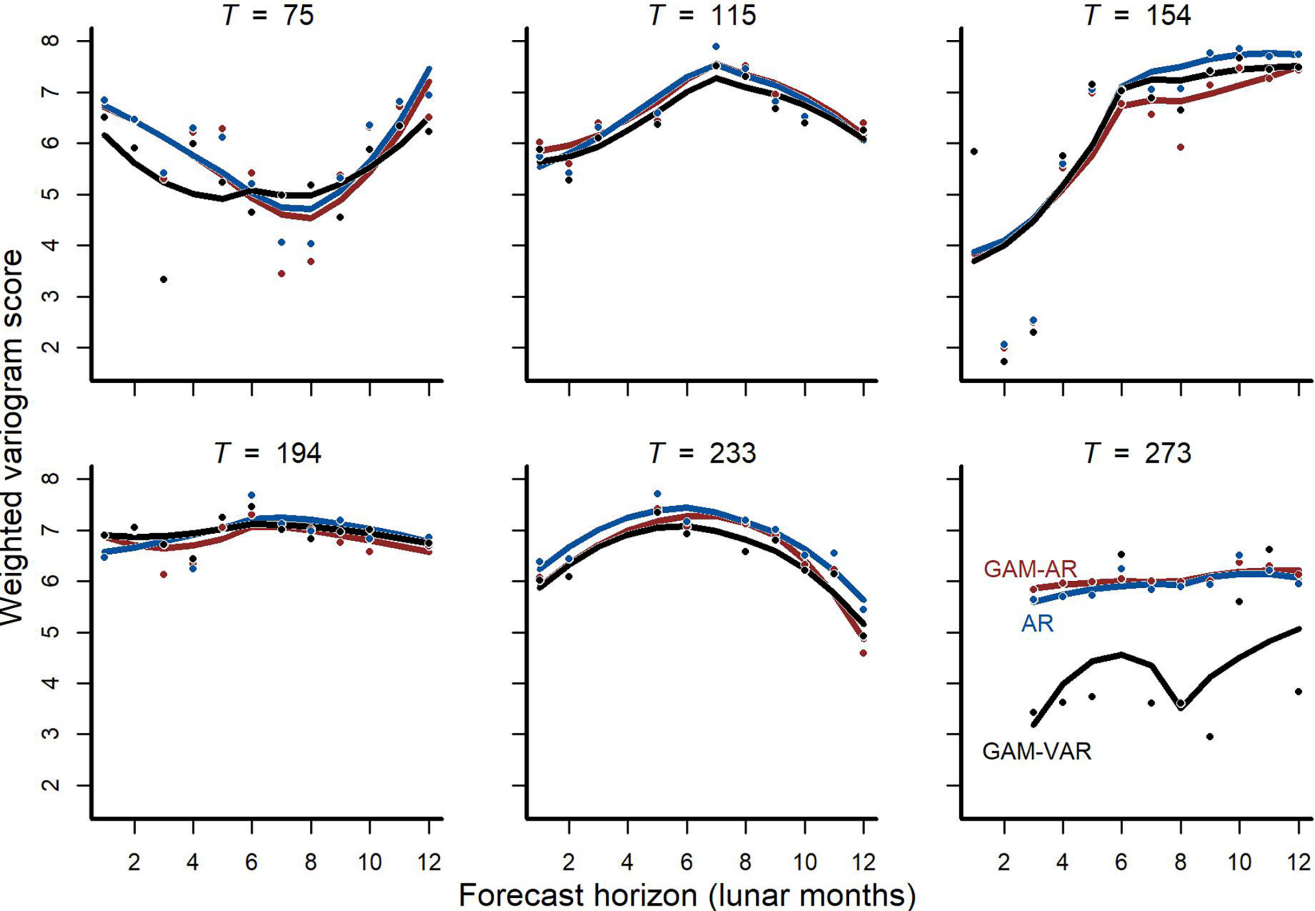
Out-of-sample validation (forecast) metrics for competing models. Cross-validation forecast performances for three of the competing models (we do not show metrics for the *GAM-AR no pooling* model as they were not clearly distinguishable from the *GAM-AR* metrics). Y-axis shows the log of the weighted variogram score, a scoring rule that penalizes multivariate forecasts if they are not well calibrated and do not capture inter-series correlations in observed data (lower scores are preferred). A total of 12-step ahead predictions were evaluated over a sequence of six evenly spaced origins. Points show individual forecast scores, with lower scores indicating a better forecast. Lines show Loess smoothed trend lines. Missing points indicate that sampling did not occur at the time point for that horizon.

We also found that forecast performance differed among models, with more complex multi-species models again tending to score higher for forecast performance than simpler models. Forecasts from the multi-species *GAM-VAR* were the most accurate when considering all validation points in aggregate and for 4/6 cross-validation folds (Fig. 3; Fig. S8). The *GAM-AR* and *GAM-AR no pooling* models gave similar predictions and effectively tied for second in forecast performance, giving the most accurate forecasts in 2/6 cross-validation folds (Fig. 3). The simplest *AR* model gave the worst forecasts.

**Figure 4:**
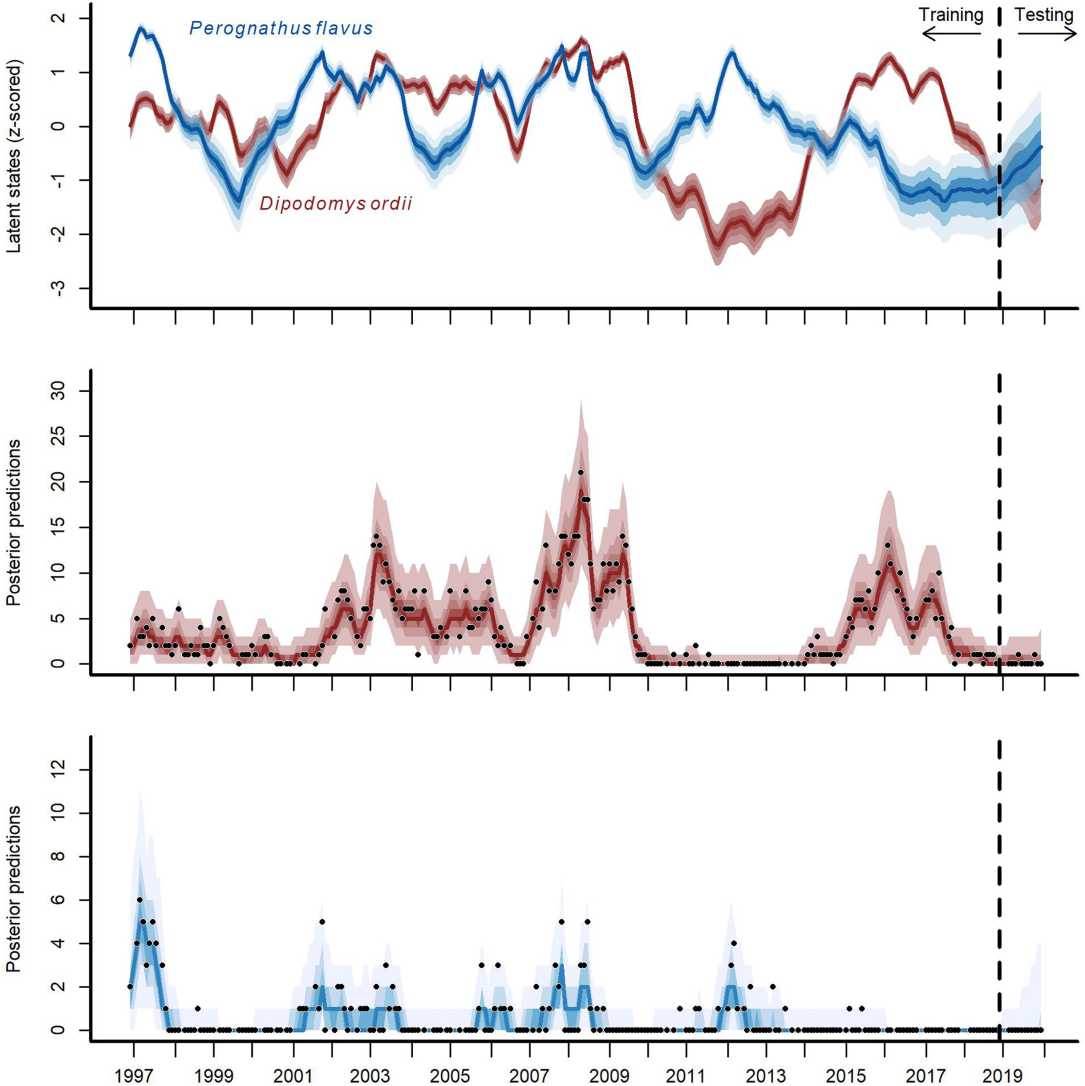
Hindcast and forecast predictions for two competing rodent species. Posterior latent state estimates (top panel) and posterior predictions (bottom two panels) from the *GAM-VAR* model for Ord’s kangaroo rat (*Dipodomys ordii*; in red) and silky pocket mouse (*Perognathus flavus*; in blue) for the training and testing periods (demarked by the vertical dashed line). State estimates were scaled to unit variance for comparisons. Ribbon shading shows posterior empirical quantiles (90^th^, 60^th^, 40^th^ and 20^th^). Dark lines show posterior medians. Points show observations.

The multi-species *GAM-VAR* model estimated large, positive autoregressive coefficients for most species (diagonal entries in Fig. S9). It also relied strongly on information from multiple species by estimating many non-zero cross-dependence effects (off-diagonal entries in Fig. S9) and process error correlations (Fig. S10), which provided structure that the model leveraged to accurately simulate historical dynamics. The model recovered multiple notable transitions observed in the time-series including a major shift in community composition around the year 2000 following the establishment of Bailey’s pocket mouse *C. baileyi*, and a second restructuring that happened following a drought in 2008–09 (Fig. S11). It was these multi-species effects that enabled the *GAM-VAR* to produce more accurate forecasts compared to the benchmarks. For example, Ord’s kangaroo rat (*Dipodomys ordii*) and silky pocket mouse (*Perognathus flavus*) had negative cross-dependencies in the *GAM-VAR*, providing additional information that could be used to make more precise predictions (Fig. 4). The benchmarks, which ignored this structure, produced smoother, less synchronous trends and wider uncertainties (Fig. S12). In the following sections, we use simulations to briefly interpret each of the multi-species effects that allowed the *GAM-VAR* to outperform simpler models.

### Modeling relationships among species provides unique insights into community dynamics

**Figure 5:**
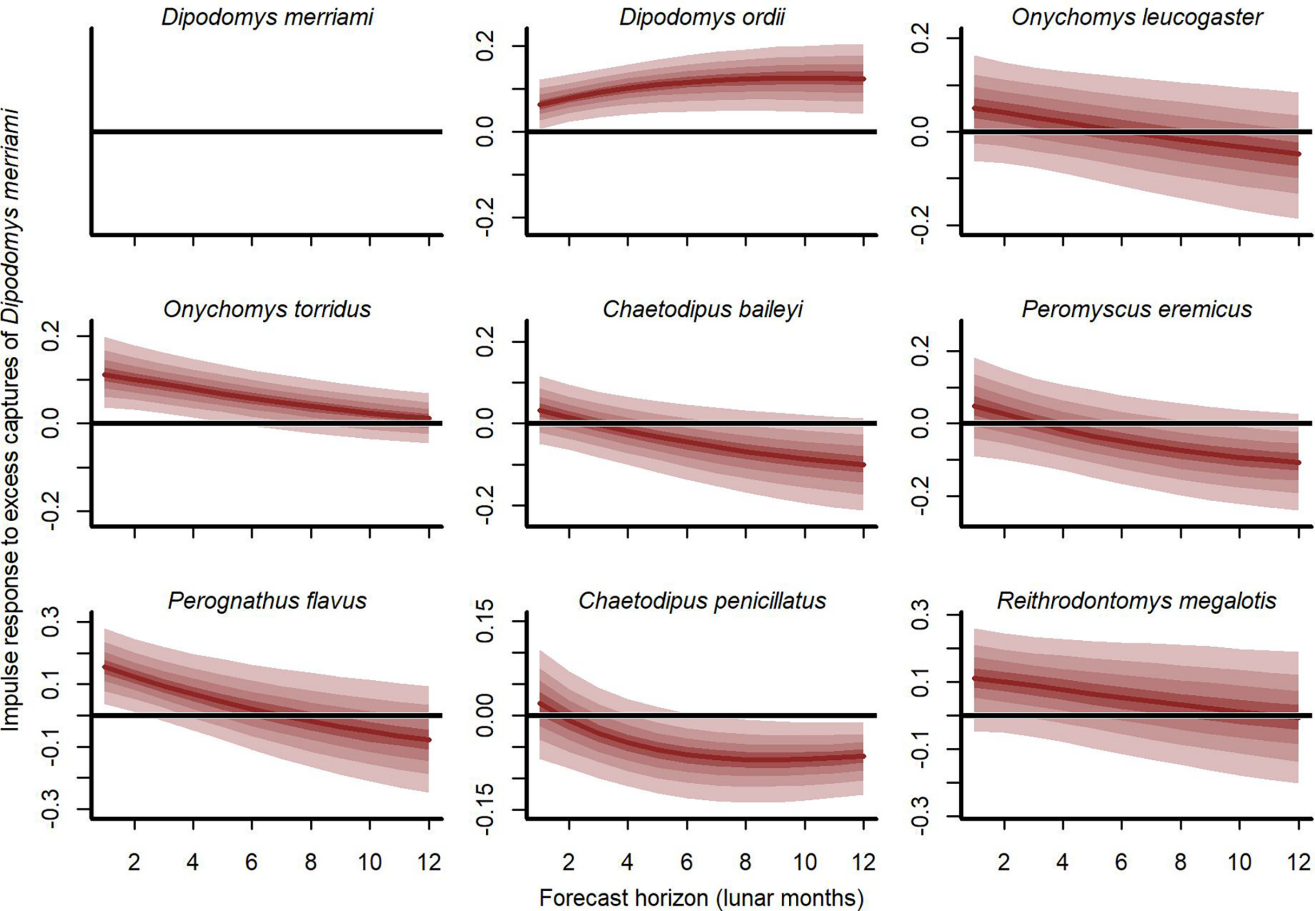
Expected responses to a simulated pulse in captures of Merriam’s kangaroo rat (*D. merriami*). Ribbon plots show how mean captures (*µ*, on the log scale) are expected to change over the next 6 months if three additional *D. merriami* individuals are captured. Ribbons show posterior empirical quantiles (90^th^, 60^th^, 40^th^ and 20^th^). Dark red lines show posterior medians.

Our cross-validation metrics strongly favoured the *GAM-VAR* and suggested that the multivariate dynamic component was a particularly important driver of increased performance. Estimates of process error were larger for the benchmarks than the *GAM-VAR* for nearly all species (Fig. S13), suggesting this model used additional information from multi-species cross-dependencies to produce better predictions. But interpreting this cross-dependence is difficult because VAR effects provide only a marginal view into the complex network of conditional associations. We therefore used impulse response functions (Lütkepohl, 1990) to better understand the model. These functions involve simulating an ‘impulse’ in captures for one species and then evaluating how predicted captures for other species changed over the next 6 months (Fig. 5). Following a simulated impulse of three extra captures for Merriam’s kangaroo rat (*D. merriami*), the model expected some initial increases (due to the correlated process errors) followed by declines in captures for most of the other species (Fig. 5). The shapes of these declines varied by species. Captures for the two pocket mouse species (*C. baileyi* and *C. penicillatus*) showed more immediate declines, while the two grasshopper mouse species (*O. leucogaster* and *O. torridus*) declined more gradually (Fig. 5). In contrast, the other kangaroo rat species (*D. ordii*) was expected to increase following a *D. merriami* pulse (Fig. 5). Different effects were expected when changing the focal species (Fig. S14).

### Positive NDVI associations and hierarchical minimum temperature effects

**Figure 6:**
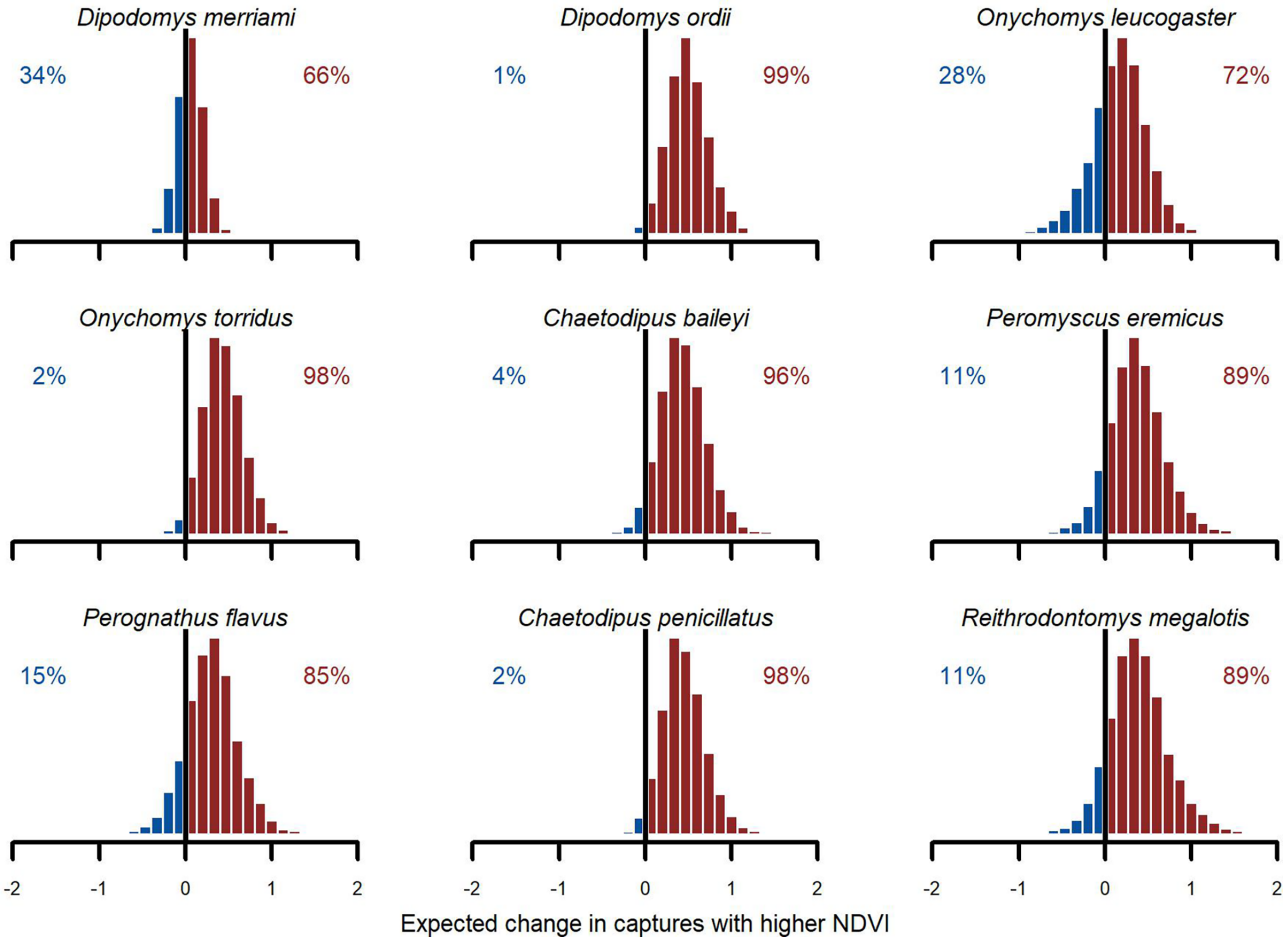
Posterior NDVI contrasts from the hierarchical slopes component of the *GAM-VAR* model. Histograms illustrate how much the expected number of captures, *exp (*µ*)*, would change if the z-scored NDVI 12-month moving average changed from a relatively low value (−0.50) to a relatively high value (0.50). Numbers in each plot indicate the proportion of probability mass at or below zero (in blue) *vs*. above zero (in red).

We found broad support for positive associations with $NDV{I_{MA12}}$ (Fig. 6). Conditional simulations, which asked how rodents might respond if moved from a relatively dry/brown vegetation state to a relatively moist/green vegetation state, gave higher probability to increased captures in the moist/green scenario for all species. But uncertainties about this effect varied among species. Greatest increases were expected for Ord’s kangaroo rat (*D. ordii*), Western harvest mouse (*R. megalotis*) and cactus mouse (*Peromyscus eremicus*). The model was less confident about the direction of effect for Northern grasshopper mouse (*O. leucogaster*) and for one of the most dominant species in the study, Meriam’s kangaroo rat (*D. merriami*). For these species, the model showed a ~70% chance of increasing abundance in the higher $NDV{I_{MA12}}$ scenario (Fig. 6). While primary conclusions were generally similar when using the *GAM-AR no pooling* model, which did not leverage multi-species learning, the estimates of these contrasts were much more variable.

Interpreting minimum temperature distributed lag effects also required simulation. We visualized 1,000 simulated functions for each species using temperatures from the year 1997 (Fig. S15). There was large uncertainty in function shapes for all species except the desert pocket mouse (*C. penicillatus*). Captures for this species were expected to increase from May to October and decrease sharply in winter. For seven of the other eight species, the model generally expected more captures in spring (March–May) and fewer in late summer/autumn (July–October). But the shapes of these responses varied. The two kangaroo rats (*D. merriami* and *D. ordii*) had smoother shapes that decreased gradually from mid-summer to mid-winter. But the model expected *D. ordii* captures to peak slightly later (May as opposed to March for *D. merriami*). The Southern grasshopper mouse (*O. torridus*) was the only species that was expected to show higher captures in late autumn/early winter (Fig. S16). The five species that relied solely on the global distributed lag minimum temperature function (*O. leucogaster*, *C. baileyi*, *P. eremicus*, *P. flavus* and *R. megalotis*) were expected to show tighter spring peaks (higher captures in April and May) and autumn troughs (fewer captures in August and September). When simulating from the *GAM-AR no pooling* model, the lack of multi-species learning was immediately obvious. There was not enough information to learn nonlinear distributed lag functions for these five species, with the model instead estimating flat functions centred on zero for all five species (Fig. S16).

## Discussion

Understanding and predicting change in species abundances requires models that capture realistic biotic structure and that can address data complexities to produce near-term ecological forecasts (Hampton et al., 2013; Holmes, Ward & Scheuerell, 2014). Our results show that incorporating relationships between species to estimate their lagged dependence, and to learn their potentially non-linear associations with environmental drivers, yields more accurate in-sample and out-of-sample predictions. In addition to improved quantitative forecasts, incorporating these multi-species complexities provides deeper insights into the dynamics of the system that could be important for scenario planning and other qualitative forecasting approaches. For example, our dynamic VAR process uncovered biotic structure representing a cascading network of relationships within the Portal rodent community. Captures for all species increased with higher NDVI and responded nonlinearly to temperature change, but the shapes and magnitudes of these responses differed across species. Our results show that models that describe biological complexity, both through nonlinear covariate functions and multi-species dependence, are useful both for generating more accurate near-term forecasts and for asking targeted questions about drivers of ecological change (Greenville et al., 2016; Ives et al., 2003; Ovaskainen et al., 2017; Pedersen et al., 2019).

### Leveraging relationships between species for ecological forecasting

Interactions and dependencies among multiple species are hypothesized to play pivotal roles in the assembly of ecological communities and for broader ecosystem functions (Dobzhansky, 1950; Fecchio et al., 2019; Mayfield & Stouffer, 2017; Mutshinda, O’Hara & Woiwod, 2009). This study shows why models that target multi-species effects in both their environmental responses and their biotic dependencies should also be strongly considered when studying community dynamics. Our approach to constructing hierarchical dynamic GAMs and evaluating forecasts using multivariate proper scoring rules offers a way to quantitatively assess multi-species forecasts and scrutinize their value in real-world ecological forecasting applications. We also demonstrate how inferences from these models provide deeper insights into why they may or may not perform better. For example, the *GAM-VAR*’s process error estimates were smaller than those from the benchmarks because it used more information from the data. By learning about the relationships between species the model could better capture both shared responses to environmental factors (*e.g*., a wet year in the desert is good for most species) and direct temporal effects (*e.g*., dynamic competition for seeds). These relationships between species can allow forecasts for less common species to borrow strength from more common species, yielding better hindcasts and forecasts compared to simpler single-species models. But like other multivariate autoregressive models (Hannaford et al., 2023; Holmes, Ward & Scheuerell, 2014; Ives et al., 2003) the VAR parameters of the *GAM-VAR* should not be interpreted as a species interaction matrix, because these relationships can result from multiple sources (*i.e*., shared environmental responses, shared biotic responses and/or direct interactions). While the parameters are not interpretable as direct interactions, this approach does make it possible to gain a more detailed understanding of population dynamics. Conducting simulations from this type of model allows exploring which species have the strongest cascading effects, what changes might we expect if management increases or decreases abundance for target species, and how these effects relate to regime transitions.

Our hierarchical modelling approach also makes it possible to partition variance into contributions from observation error, environmental responses, and multi-species dependence to guide future efforts to improve ecological forecasting. In our study, forecasts were dominated by uncertainty in the dynamic process model, but using a Vector Autoregressive process allowed us to dissect this uncertainty in meaningful ways (Ives et al., 2003; Lütkepohl, 1990). Simulated responses to sudden impulses in captures were often delayed and nonlinear. Despite the restriction to a VAR of lag of 1 month, these responses resulted in cascading changes that lasted up to 6 months. Our model’s ability to simulate and dissect community change in this way offers a useful avenue for ecologists to better understand, and expand on, theoretical predictions from both classical and more recent empirical studies that have described strong interspecific interactions in ecological systems (Dumandan et al., 2024; Ebersole, 1977; Mayfield & Stouffer, 2017; Volterra, 1928).

### Learning hierarchical nonlinear effects from community data

Our model captured linear, nonlinear, and lagged responses to environmental and climatic covariates that were informed by data from all species at once. We found positive linear associations between capture rates and a 12-month moving average of NDVI. This response was expected because the rodents at Portal depend on plants for food and other resources (Brown & Ernest, 2002; Ernest et al., 2020) and NDVI measures vegetation greenness in the landscape. But within this overarching community pattern there were interesting patterns of variation in these responses among species. The strongest positive association was shown by Ord’s kangaroo rat (*D. ordii*), a species that field evidence suggests consumes and harvests grasses (Kerley, Whitford & Kay, 1997). In contrast, Merriam’s kangaroo rat (*D. merriami*) showed weaker associations with NDVI. This species has been predicted to increase in relative abundance in the study region with more severe droughts, in part due to a preference for more open foraging habitat with less vegetation (Cárdenas et al., 2021).

Distributed lag functions determine the best combination of lags for environmental covariates but are not commonly used in ecology (but see Karunarathna, Wells & Clark, 2024; Ogle et al., 2015; Wells et al., 2016). Our study shows how these effects can be learned hierarchically and provides useful insights into delayed responses to temperature change for rodent species at Portal. Most species showed higher captures when minimum temperatures were low 3–4 months prior, suggesting increases begin during mid to late spring when resources such as seeds become available. But others, such as Merriam’s kangaroo rat and Southern grasshopper mouse, showed increases during cooler months in autumn and winter. Asynchronous phenology, where species show different reproductive timing, is sometimes expected in competitive communities (Carter & Rudolf, 2022). Analysis of individual reproductive status in different biotic contexts suggests that some species shift their reproductive timing in the presence of strong competitors in the Portal system (Dumandan, Yenni & Ernest, 2023). Do these competitive forces play a role in seasonal capture variation over the long-term? Comparing temperature responses on control *vs*. experimental plots would be one interesting way to tackle this question.

Interestingly, despite the relatively large number of observations our data contained for each species, estimates of environmental responses were still more precise and informative when using hierarchical models (which use partial pooling) as opposed to a no-pooling model that only considers species’ effects in isolation. While hierarchical intercepts and slopes are commonly used in ecological models, there has been less emphasis on hierarchical nonlinear functions (but see Pedersen et al., 2019). Open access to new software that makes it easy to construct and estimate these types of functions, such as the *mvgam* R package that we used here (Clark & Wells, 2023), should improve their uptake in ecological forecasting exercises.

But despite the power of hierarchical environmental effects to improve predictions, we cannot interpret environmental response estimates as directly causal for several reasons. First, we know NDVI is not a perfect measure of changes in seed production. Second, it is likely that changes to NDVI and minimum temperature are both related to other unmeasured environmental drivers that may also influence rodent abundance. Major storms, the El Niño Southern Oscillation and other factors that influence moisture levels can all influence temperature and vegetation change (Sun & Kafatos, 2007). These other drivers could act as unmeasured confounds, biasing estimates in a causal inference framework (McElreath, 2020).

### Future directions

Two additional steps would be useful to fully assess the value of multi-species models for ecological forecasting, both in this system and more broadly as an ecological application. First, a more diverse suite of candidate models could be estimated to determine how forecasts could be combined into an ensemble to provide the best predictions in situations where prediction accuracy is the primary goal (Clark et al., 2022; Powell-Romero et al., 2023). This could be especially useful for detecting changes in the system. For example, *GAM-VAR* gave better forecasts in most cross-validation tests, but its performance was slightly worse than the simpler *GAM-AR* when the training window stopped just prior to a major restructuring of rodent abundances that was taking place in response to a drought. Second, determining which models are best for true forecasting requires evaluating forecasts in the presence of uncertainty in future covariate values. In this study we were hindcasting and therefore used the actual observed environmental measurements for the period reserved for model evaluation. Fortunately, the system is undergoing active forecasting involving a suite of simpler models and leveraging actual forecasts for environmental covariates (Simonis et al., 2022; White et al., 2019). A natural next step for this work is to compare the performance of the *GAM-VAR* model to simpler models both using hindcasting with observed covariates and when making true forecasts relying on predictions instead of observations for NDVI and minimum temperature.

The Portal Project also provides a unique opportunity to disentangle the combined influence of shared environmental responses and direct species interactions in driving observed relationships between species. The site includes a long-term experimental manipulation where kangaroo rats (*Dipodomys* species) are excluded from some plots. Recent work shows that single species forecasting models for *C. baileyi* do not transfer well between the control plots and this experimental manipulation, likely due to the different competitive environment experienced in the absence of the behaviorially dominant kangaroo rats (Dumandan, Yenni & Ernest, 2023). Multi-species models like the *GAM-VAR* have the potential to transfer better in situations where one or more species are removed from the system by accurately predicting the response of the other species to this removal. Therefore, a key next step in evaluating the potential strengths of our models is to determine if they can more effectively transfer to make accurate predictions on the plots with the experimentally manipulated species composition. More broadly, we hope that the ability to estimate multi-species dependence and species-level variation in nonlinear environmental responses result in more accurate forecasts, inspire new questions, and lead to an improved understanding of the factors that govern ecological community dynamics.

## Supplemental Information

10.7717/peerj.18929/supp-1 Supplemental Information 1 Total rodent captures from the Portal Project for the period December 1996 to August 2022. Counts represent total captures for nine species across long-term control plots, sampled at each cycle of the lunar moon. Blanks represent missing values.

10.7717/peerj.18929/supp-2 Supplemental Information 2 Autocorrelation functions of rodent capture time series in the Portal Project. Dashed lines show values beyond which the autocorrelations are considered significantly different from zero.

10.7717/peerj.18929/supp-3 Supplemental Information 3 Histograms of rodent capture time series in the Portal Project. Counts represent total captures across long-term control plots, sampled at each cycle of the lunar moon.

10.7717/peerj.18929/supp-4 Supplemental Information 4 Seasonal and Trend decomposition using Loess smoothing (STL) applied to observed minimum temperature time series for the period December 1996 – August 2022. The top panel shows the raw time series. The middle plot shows the estimated long-term trend (calculated using a Loess regression to the de-seasoned time series). The bottom plot shows the time-varying estimate of seasonality (calculated using a Loess regression that smooths across years).

10.7717/peerj.18929/supp-5 Supplemental Information 5 Temporal observations of Normalized Difference Vegetation Index (NDVI) in the study site. Top panel: observed Normalized Difference Vegetation Index (NDVI) time series for the period December 1996 – August 2022, with obvious seasonal fluctuations. Bottom panel: a 12-month moving average that represents smooth, gradual changes in NDVI at the study site.

10.7717/peerj.18929/supp-6 Supplemental Information 6 Autocorrelation functions of randomized quantile residuals from the *GAM-VAR* model. Ribbon shading shows posterior empirical quantiles (90^th^, 60^th^, 40^th^ and 20^th^). Dark red lines show posterior medians. Dashed lines show values beyond which the autocorrelations would be considered significantly different from zero in a Frequentist paradigm.

10.7717/peerj.18929/supp-7 Supplemental Information 7 Normal quantile-quantile plots of randomized quantile residuals from the *GAM-VAR* model. Ribbon shading shows posterior empirical quantiles (90^th^, 60^th^, 40^th^ and 20^th^). Dark lines show posterior medians.

10.7717/peerj.18929/supp-8 Supplemental Information 8 Posterior predictions from the *GAM-VAR* model for the training and testing periods (demarked by the vertical dashed line). Latent state estimates were scaled to unit variance for comparisons. Ribbon shading shows posterior empirical quantiles (90^th^, 60^th^, 40^th^ and 20^th^). Dark lines show posterior medians. Points show observations.

10.7717/peerj.18929/supp-9 Supplemental Information 9 Posterior distributions of vector autoregressive coefficients (matrix *A*). Off-diagonals represent cross-dependencies. For example, the entry in captures the effect of **DO**’s state at time t - 1 on the current state estimate for **DM** (at time t). Diagonals (with grey shading) represent autoregressive coefficients (the effect of a species’ state at time t on its own state at time t - 1). Colours indicate the proportion of probability mass at or below zero (in blue) *vs* above zero (in red). **DO**, *Dipodomys merriami*; **DO**, *Dipodomys ordii*; **OL**, *Onychomys leucogaster*; **OT**, *Onychomys torridus*; **PB**, *Chaetodipus baileyi*; **PE**, *Peromyscus eremicus*; **PF**, *Perognathus flavus*; **PP**, *Chaetodipus penicillatus*; **RM**, *Reithrodontomys megalotis*.

10.7717/peerj.18929/supp-10 Supplemental Information 10 Posterior distributions for process error correlations (matrix C). Colours indicate the proportion of probability mass at or below zero (in blue) *vs* above zero (in red). **DO**, *Dipodomys merriami*; **DO**, *Dipodomys ordii*; **OL**, *Onychomys leucogaster*; **OT**, *Onychomys torridus*; **PB**, *Chaetodipus baileyi*; **PE**, *Peromyscus eremicus*; **PF**, *Perognathus flavus*; **PP**, *Chaetodipus penicillatus*; **RM**, *Reithrodontomys megalotis*.

10.7717/peerj.18929/supp-11 Supplemental Information 11 Simulated rodent communities. Using the ***GAM-VAR*** model’s posterior predictive distribution, we simulated communities of 200 individuals at different timepoints to investigate how well the model captured known community transitions. Colours represent different species.

10.7717/peerj.18929/supp-12 Supplemental Information 12 Posterior trend estimates from three competing models for Ord’s kangaroo rat (*Dipodomys ordii*; in red) and silky pocket mouse (*Perognathus flavus*; in blue). Trends were scaled to unit variance for comparisons. Ribbon shading shows posterior empirical quantiles (90^th^, 60^th^, 40^th^ and 20^th^). Dark lines show posterior medians.

10.7717/peerj.18929/supp-13 Supplemental Information 13 Posterior estimates of trend standard deviations from the three competing models. Estimates are the square root of diagonal parameters from the trend covariance matrix (Sigma_var_) for the ***GAM-VAR*** (black), ***GAM-AR*** (red) and ***AR*** (blue).

10.7717/peerj.18929/supp-14 Supplemental Information 14 Expected responses to a pulse in captures of the desert pocket mouse (*Chaetodipus penicillatus*). Ribbon plots show how mean captures (μ, on the log scale) are expected to change over the next six months if three additional *C. penicillatus* individuals are captured. Ribbon shading shows posterior empirical quantiles (90th, 60th, 40th and 20th). Dark red lines show posterior medians.

10.7717/peerj.18929/supp-15 Supplemental Information 15 Conditional distributed lag minimum temperature functions from the hierarchical smooth component of the *GAM-VAR* model, using temperatures observed in 1997. All other effects were ignored. Functions for *O. leucogaster*, *C. baileyi*, *P. eremicus*, *P. flavus* and *R. megalotis* were drawn solely from the global function. Functions for other species were the sum of the global function and a species-specific deviation function. Estimates were scaled to unit variance for comparisons. Ribbons show posterior empirical quantiles (90^th^, 60^th^, 40^th^ and 20^th^). Dark red lines show posterior medians.

10.7717/peerj.18929/supp-16 Supplemental Information 16 Conditional distributed lag minimum temperature functions from the independent smooth component of the *GAM-AR no pooling* model, using temperatures observed in 1997. All other effects were ignored. Functions for *O. leucogaster*, *C. baileyi*, *P. eremicus*, *P. flavus* and *R. megalotis* were drawn solely from the global function. Functions for other species were the sum of the global function and a species-specific deviation function. Estimates were scaled to unit variance for comparisons. Ribbons show posterior empirical quantiles (90^th^, 60^th^, 40^th^ and 20^th^). Dark red lines show posterior medians.

## Additional Information and Declarations

### Data Availability

The following information was supplied regarding data availability:

All data from the Portal Project is is available in processed forms using the portalr R package (Christensen et al., 2019) and at Zenodo: S. K. Morgan Ernest, Glenda M. Yenni, Ginger Allington, Ellen K. Bledsoe, Erica M. Christensen, Renata Diaz, Keith Geluso, Jacob R. Goheen, Qinfeng Guo, Edward Heske, Douglas Kelt, Joan M. Meiners, Jim Munger, Carla Restrepo, Douglas A. Samson, Michele R. Schutzenhofer, Marian Skupski, Sarah R. Supp, Katherine M. Thibault, … Thomas J. Valone. (2025). weecology/PortalData: 5.123.0 (5.123.0) [Data set]. Zenodo. https://doi.org/10.5281/zenodo.14624226.

The R code to reproduce analyses is available at GitHub and Zenodo:

- https://github.com/nicholasjclark/portal_VAR.

- Nicholas Clark. (2025). nicholasjclark/portal_VAR: v1.0 (v1.0). Zenodo. https://doi.org/10.5281/zenodo.14607006.

## References

[ref-1] Abrego N, Roslin T, Huotari T, Ji Y, Schmidt NM, Wang J, Yu DW, Ovaskainen O (2021). Accounting for species interactions is necessary for predicting how arctic arthropod communities respond to climate change. Ecography.

[ref-2] Algar AC, Kharouba HM, Young ER, Kerr JT (2009). Predicting the future of species diversity: macroecological theory, climate change, and direct tests of alternative forecasting methods. Ecography.

[ref-3] Averill C, Werbin ZR, Atherton KF, Bhatnagar JM, Dietze MC (2021). Soil microbiome predictability increases with spatial and taxonomic scale. Nature Ecology & Evolution.

[ref-4] Betancourt M (2017). A conceptual introduction to Hamiltonian Monte Carlo.

[ref-5] Bledsoe EK, Ernest SM (2019). Temporal changes in species composition affect a ubiquitous species’ use of habitat patches. Ecology.

[ref-6] Brown JH, Resetarits WJ, Bernardo J (1998). The desert granivory experiments at portal. Experimental Ecology.

[ref-7] Brown JH, Ernest SM (2002). Rain and rodents: complex dynamics of desert consumers: although water is the primary limiting resource in desert ecosystems, the relationship between rodent population dynamics and precipitation is complex and nonlinear. BioScience.

[ref-8] Bunin G (2017). Ecological communities with Lotka-Volterra dynamics. Physical Review E.

[ref-9] Carpenter B, Gelman A, Hoffman MD, Lee D, Goodrich B, Betancourt M, Brubaker M, Guo J, Li P, Riddell A (2017). Stan: a probabilistic programming language. Journal of Statistical Software.

[ref-10] Carter SK, Rudolf VH (2022). Exploring conditions that strengthen or weaken the ecological and evolutionary consequences of phenological synchrony. The American Naturalist.

[ref-11] Christensen EM, Harris DJ, Ernest S (2018). Long-term community change through multiple rapid transitions in a desert rodent community. Ecology.

[ref-12] Christensen EM, Simpson GL, Ernest S (2019). Established rodent community delays recovery of dominant competitor following experimental disturbance. Proceedings of the Royal Society B: Biological Sciences.

[ref-13] Christensen EM, Yenni GM, Ye H, Simonis JL, Bledsoe EK, Diaz R, Taylor SD, White EP, Ernest S (2019). portalr: an R package for summarizing and using the portal project data. Journal of Open Source Software.

[ref-14] Clark JS, Carpenter SR, Barber M, Collins S, Dobson A, Foley JA, Lodge DM, Pascual M, Pielke R, Pizer W (2001). Ecological forecasts: an emerging imperative. Science.

[ref-15] Clark NJ, Kerry JT, Fraser CI (2020). Rapid winter warming could disrupt coastal marine fish community structure. Nature Climate Change.

[ref-16] Clark NJ, Proboste T, Weerasinghe G, Soares Magalhães RJ (2022). Near-term forecasting of companion animal tick paralysis incidence: an iterative ensemble model. PLOS Computational Biology.

[ref-17] Clark NJ, Wells K (2023). Dynamic generalised additive models (DGAMs) for forecasting discrete ecological time series. Methods in Ecology and Evolution.

[ref-18] Clark NJ, Wells K, Dimitrov D, Clegg SM (2016). Co-infections and environmental conditions drive the distributions of blood parasites in wild birds. Journal of Animal Ecology.

[ref-19] Cárdenas PA, Christensen E, Ernest SKM, Lightfoot DC, Schooley RL, Stapp P, Rudgers JA (2021). Declines in rodent abundance and diversity track regional climate variability in North American drylands. Global Change Biology.

[ref-20] Daugaard U, Munch SB, Inauen D, Pennekamp F, Petchey OL (2022). Forecasting in the face of ecological complexity: number and strength of species interactions determine forecast skill in ecological communities. Ecology Letters.

[ref-21] Dietze MC, Fox A, Beck-Johnson LM, Betancourt JL, Hooten MB, Jarnevich CS, Keitt TH, Kenney MA, Laney CM, Larsen LG (2018). Iterative near-term ecological forecasting: needs, opportunities, and challenges. Proceedings of the National Academy of Sciences of the United States of America.

[ref-22] Dietze MC, Serbin SP, Davidson C, Desai AR, Feng X, Kelly R, Kooper R, LeBauer D, Mantooth J, McHenry K (2014). A quantitative assessment of a terrestrial biosphere model’s data needs across North American biomes. Journal of Geophysical Research: Biogeosciences.

[ref-23] Dobzhansky T (1950). Evolution in the tropics. American Scientist.

[ref-24] Dumandan PKT, Simonis JL, Yenni GM, Ernest SKM, White EP (2024). Transferability of ecological forecasting models to novel biotic conditions in a long-term experimental study. Ecology.

[ref-25] Dumandan PKT, Yenni GM, Ernest M (2023). Shifts in competitive structures can drive variation in species phenology. Ecology.

[ref-26] Ebersole JP (1977). The adaptive significance of interspecific territoriality in the reef fish *Eupomacentrus leucostictus*. Ecology.

[ref-27] Ernest S, Brown JH (2001). Delayed compensation for missing keystone species by colonization. Science.

[ref-28] Ernest S, Yenni GM, Allington G, Bledsoe EK, Christensen EM, Diaz RM, Geluso K, Goheen JR, Guo Q, Heske E, Kelt D, Meiners JM, Munger J, Restrepo C, Samson DA, Schutzenhofer MR, Skupski M, Supp SR, Thibault KM, Taylor SD, White EP, Ye H, Davidson DW, Brown JH, Valone TJ (2020). The portal project: a long-term study of a chihuahuan desert ecosystem.

[ref-29] Fecchio A, Wells K, Bell JA, Tkach VV, Lutz HL, Weckstein JD, Clegg SM, Clark NJ (2019). Climate variation influences host specificity in avian malaria parasites. Ecology Letters.

[ref-30] Fredston AL, Cheung WWL, Frölicher TL, Kitchel ZJ, Maureaud AA, Thorson JT, Auber A, Mérigot B, Palacios-Abrantes J, Palomares MLD, Pecuchet L, Shackell NL, Pinsky ML (2023). Marine heatwaves are not a dominant driver of change in demersal fishes. Nature.

[ref-31] Gabry J, Češnovar R (2021). Cmdstanr: R interface to ‘CmdStan’. https://mc-stan.org/cmdstanr.

[ref-32] Gerber LR, Botsford LW, Hastings A, Possingham HP, Gaines SD, Palumbi SR, Andelman S (2003). Population models for marine reserve design: a retrospective and prospective synthesis. Ecological Applications.

[ref-33] Greenville AC, Wardle GM, Nguyen V, Dickman CR (2016). Population dynamics of desert mammals: similarities and contrasts within a multispecies assemblage. Ecosphere.

[ref-34] Hampton SE, Holmes EE, Scheef LP, Scheuerell MD, Katz SL, Pendleton DE, Ward EJ (2013). Quantifying effects of abiotic and biotic drivers on community dynamics with multivariate autoregressive (MAR) models. Ecology.

[ref-35] Hannaford NE, Heaps SE, Nye TM, Curtis TP, Allen B, Golightly A, Wilkinson DJ (2023). A sparse Bayesian hierarchical vector autoregressive model for microbial dynamics in a wastewater treatment plant. Computational Statistics & Data Analysis.

[ref-36] Harris DJ, Taylor SD, White EP (2018). Forecasting biodiversity in breeding birds using best practices. PeerJ.

[ref-37] Heaps SE (2023). Enforcing stationarity through the prior in vector autoregressions. Journal of Computational and Graphical Statistics.

[ref-38] Heske EJ, Brown JH, Mistry S (1994). Long-term experimental study of a Chihuahuan Desert rodent community: 13 years of competition. Ecology.

[ref-39] Holmes E, Ward E, Scheuerell M (2014). Analysis of multivariate time-series using the MARSS package. NOAA Fisheries, Northwest Fisheries Science Center.

[ref-40] Ibáñez I, Silander JA, Wilson AM, LaFleur N, Tanaka N, Tsuyama I (2009). Multivariate forecasts of potential distributions of invasive plant species. Ecological Applications.

[ref-41] IPBES (2019). Global assessment report on biodiversity and ecosystem services of the Intergovernmental Science-Policy Platform on Biodiversity and Ecosystem Services.

[ref-42] Ives AR, Abbott KC, Ziebarth NL (2010). Analysis of ecological time series with ARMA (p, q) models. Ecology.

[ref-43] Ives AR, Dennis B, Cottingham KL, Carpenter SR (2003). Estimating community stability and ecological interactions from time-series data. Ecological Monographs.

[ref-44] Johnson-Bice SM, Ferguson JM, Erb JD, Gable TD, Windels SK (2021). Ecological forecasts reveal limitations of common model selection methods: predicting changes in beaver colony densities. Ecological Applications.

[ref-45] Karp MA, Link JS, Grezlik M, Cadrin S, Fay G, Lynch P, Townsend H, Methot RD, Adams GD, Blackhart K (2023). Increasing the uptake of multispecies models in fisheries management. ICES Journal of Marine Science.

[ref-46] Karunarathna KANK, Wells K, Clark NJ (2024). Modelling nonlinear responses of a desert rodent species to environmental change with hierarchical dynamic generalized additive models. Ecological Modelling.

[ref-47] Kerley GI, Whitford WG, Kay FR (1997). Mechanisms for the keystone status of kangaroo rats: graminivory rather than granivory?. Oecologia.

[ref-48] Lewis AS, Rollinson CR, Allyn AJ, Ashander J, Brodie S, Brookson CB, Collins E, Dietze MC, Gallinat AS, Juvigny-Khenafou N (2023). The power of forecasts to advance ecological theory. Methods in Ecology and Evolution.

[ref-49] Lewis ASL, Woelmer WM, Wander HL, Howard DW, Smith JW, McClure RP, Lofton ME, Hammond NW, Corrigan RS, Thomas RQ, Carey CC (2022). Increased adoption of best practices in ecological forecasting enables comparisons of forecastability. Ecological Applications.

[ref-50] Lima M, Ernest SM, Brown JH, Belgrano A, Stenseth NC (2008). Chihuahuan Desert kangaroo rats: nonlinear effects of population dynamics, competition, and rainfall. Ecology.

[ref-51] Lindenmayer DB, Likens GE, Andersen A, Bowman D, Bull CM, Burns E, Dickman CR, Hoffmann AA, Keith DA, Liddell MJ (2012). Value of long-term ecological studies. Austral Ecology.

[ref-52] Lütkepohl H (1990). Asymptotic distributions of impulse response functions and forecast error variance decompositions of vector autoregressive models. The Review of Economics and Statistics.

[ref-53] Mayfield MM, Stouffer DB (2017). Higher-order interactions capture unexplained complexity in diverse communities. Nature Ecology & Evolution.

[ref-54] McElreath R (2020). Statistical rethinking: a bayesian course with examples in R and stan.

[ref-55] Moustahfid H, Hendrickson LC, Arkhipkin A, Pierce GJ, Gangopadhyay A, Kidokoro H, Markaida U, Nigmatullin C, Sauer WH, Jereb P (2021). Ecological-fishery forecasting of squid stock dynamics under climate variability and change: review, challenges, and recommendations. Reviews in Fisheries Science & Aquaculture.

[ref-56] Mutshinda CM, O’Hara RB, Woiwod IP (2009). What drives community dynamics?. Proceedings of the Royal Society B: Biological Sciences.

[ref-57] Norberg A, Abrego N, Blanchet FG, Adler FR, Anderson BJ, Anttila J, Araújo MB, Dallas T, Dunson D, Elith J, Foster SD, Fox R, Franklin J, Godsoe W, Guisan A, O’Hara B, Hill NA, Holt RD, Hui FKC, Husby M, Kålås JA, Lehikoinen A, Luoto M, Mod HK, Newell G, Renner I, Roslin T, Soininen J, Thuiller W, Vanhatalo J, Warton D, White M, Zimmermann NE, Gravel D, Ovaskainen O (2019). A comprehensive evaluation of predictive performance of 33 species distribution models at species and community levels. Ecological Monographs.

[ref-58] Ogle K, Barber JJ, Barron-Gafford GA, Bentley LP, Young JM, Huxman TE, Loik ME, Tissue DT (2015). Quantifying ecological memory in plant and ecosystem processes. Ecology Letters.

[ref-59] Ovaskainen O, Tikhonov G, Dunson D, Grøtan V, Engen S, Sæther B-E, Abrego N (2017). How are species interactions structured in species-rich communities? A new method for analysing time-series data. Proceedings of the Royal Society B: Biological Sciences.

[ref-60] Paniw M, García-Callejas D, Lloret F, Bassar RD, Travis J, Godoy O (2023). Pathways to global-change effects on biodiversity: new opportunities for dynamically forecasting demography and species interactions. Proceedings of the Royal Society B.

[ref-61] Pedersen EJ, Miller DL, Simpson GL, Ross N (2019). Hierarchical generalized additive models in ecology: an introduction with mgcv. PeerJ.

[ref-62] Powell-Romero F, Fountain-Jones NM, Norberg A, Clark NJ (2023). Improving the predictability and interpretability of co-occurrence modelling through feature-based joint species distribution ensembles. Methods in Ecology and Evolution.

[ref-63] Quinn TJ (2003). Ruminations on the development and future of population dynamics models in fisheries. Natural Resource Modeling.

[ref-64] R Core Team (2023). R: a language and environment for statistical computing.

[ref-65] Romañach SS, Haider SM, Hackett C, McKelvy M, Pearlstine LG (2022). Managing multiple species with conflicting needs in the greater everglades. Ecological Indicators.

[ref-66] Royle JA, Nichols JD (2003). Estimating abundance from repeated presence-absence data or point counts. Ecology.

[ref-67] Ruiz-Moreno A, Emslie MJ, Connolly SR (2024). High response diversity and conspecific density-dependence, not species interactions, drive dynamics of coral reef fish communities. Ecology Letters.

[ref-68] Sandal L, Grøtan V, Sæther B-E, Freckleton RP, Noble DG, Ovaskainen O (2022). Effects of density, species interactions, and environmental stochasticity on the dynamics of British bird communities. Ecology.

[ref-69] Scheuerer M, Hamill TM (2015). Variogram-based proper scoring rules for probabilistic forecasts of multivariate quantities. Monthly Weather Review.

[ref-70] Simonis JL, White EP, Ernest SKM (2021). Evaluating probabilistic ecological forecasts. Ecology.

[ref-71] Simonis JL, Yenni GM, Bledsoe EK, Christensen EM, Senyondo H, Taylor SD, Ye H, White EP, Ernest SM (2022). portalcasting: supporting automated forecasting of rodent populations. Journal of Open Source Software.

[ref-72] Stan Development Team (2022). Stan modeling language users guide and reference manual, version 2.26.1. https://mc-stan.org.

[ref-73] Sun D, Kafatos M (2007). Note on the NDVI-LST relationship and the use of temperature-related drought indices over North America. Geophysical Research Letters.

[ref-74] Thorson JT, Ianelli JN, Larsen EA, Ries L, Scheuerell MD, Szuwalski C, Zipkin EF (2016). Joint dynamic species distribution models: a tool for community ordination and spatio-temporal monitoring. Global Ecology and Biogeography.

[ref-75] Tobler MW, Kéry M, Hui FK, Guillera-Arroita G, Knaus P, Sattler T (2019). Joint species distribution models with species correlations and imperfect detection. Ecology.

[ref-76] Tonkin JD, Bogan MT, Bonada N, Rios-Touma B, Lytle DA (2017). Seasonality and predictability shape temporal species diversity. Ecology.

[ref-77] Tulloch AI, Hagger V, Greenville AC (2020). Ecological forecasts to inform near-term management of threats to biodiversity. Global Change Biology.

[ref-78] Vehtari A, Gelman A, Gabry J (2017). Practical Bayesian model evaluation using leave-one-out cross-validation and WAIC. Statistics and Computing.

[ref-79] Volterra V (1928). Variations and fluctuations of the number of individuals in animal species living together. ICES Journal of Marine Science.

[ref-80] Ward EJ, Chirakkal H, González-Suárez M, Aurioles-Gamboa D, Holmes EE, Gerber L (2010). Inferring spatial structure from time‐series data: using multivariate state‐space models to detect metapopulation structure of California sea lions in the Gulf of California, Mexico. Journal of Applied Ecology.

[ref-81] Ward EJ, Holmes EE, Thorson JT, Collen B (2014). Complexity is costly: a meta-analysis of parametric and non-parametric methods for short-term population forecasting. Oikos.

[ref-82] Ward EJ, Marshall K, Scheuerell MD (2022). Regularizing priors for Bayesian VAR applications to large ecological datasets. PeerJ.

[ref-83] Warton DI, Blanchet FG, O’Hara RB, Ovaskainen O, Taskinen S, Walker SC, Hui FK (2015). So many variables: joint modeling in community ecology. Trends in Ecology & Evolution.

[ref-84] Wells K, O’Hara RB, Cooke BD, Mutze GJ, Prowse TAA, Fordham DA (2016). Environmental effects and individual body condition drive seasonal fecundity of rabbits: identifying acute and lagged processes. Oecologia.

[ref-85] White EP, Yenni GM, Taylor SD, Christensen EM, Bledsoe EK, Simonis JL, Ernest S (2019). Developing an automated iterative near-term forecasting system for an ecological study. Methods in Ecology and Evolution.

