# Supplementary figures and images for: Beyond single-species models: leveraging multispecies forecasts to navigate the dynamics of ecological predictability
Source: PeerJ. 2025 Feb 19;13:e18929. doi: 10.7717/peerj.18929 (PMC11846506; doi:10.7717/peerj.18929)

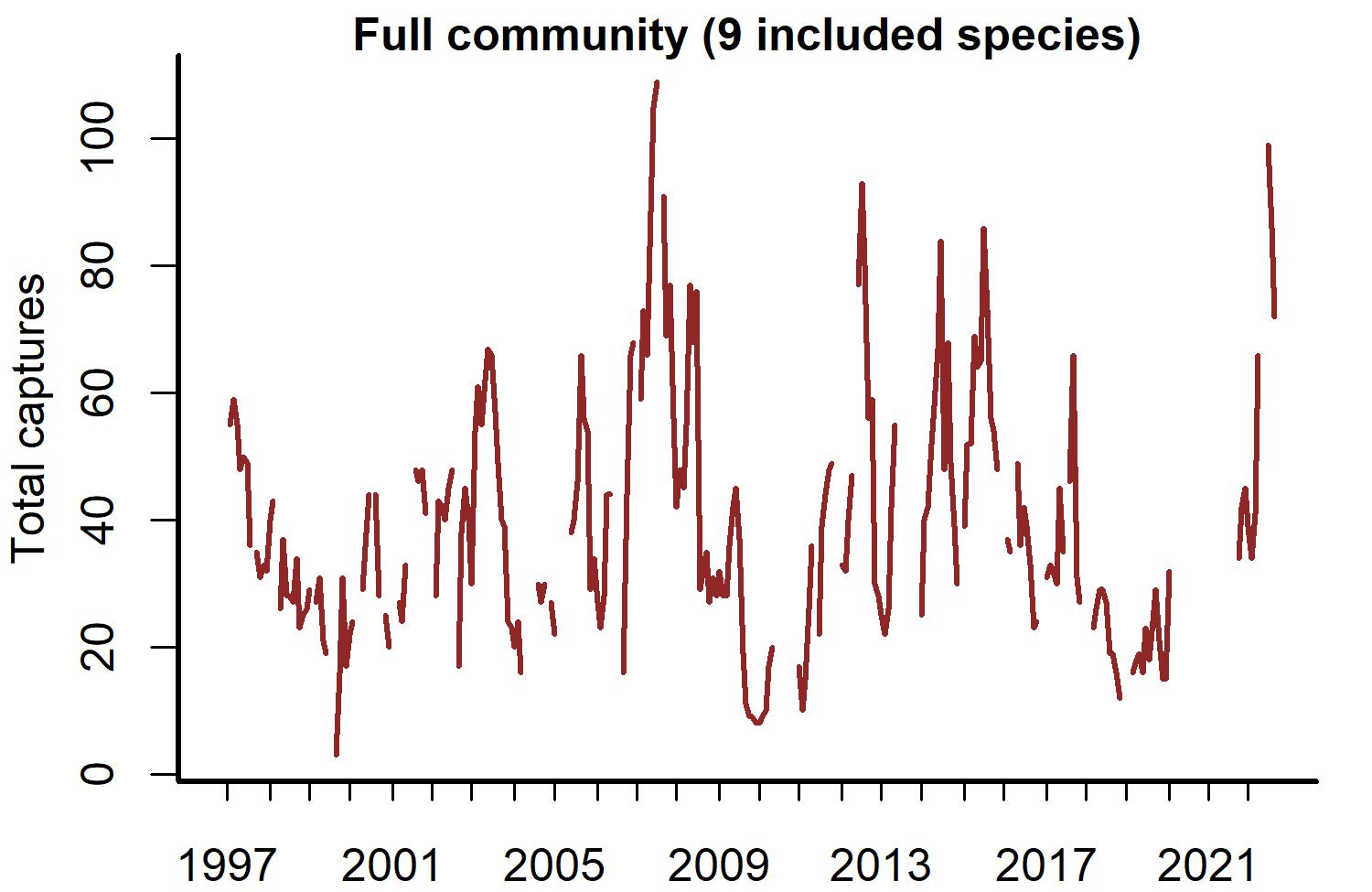

Supplement: Supplemental Information 1 — Counts represent total captures for nine species across long-term control plots, sampled at each cycle of the lunar moon. Blanks represent missing values. [file peerj-13-18929-s001.jpg]

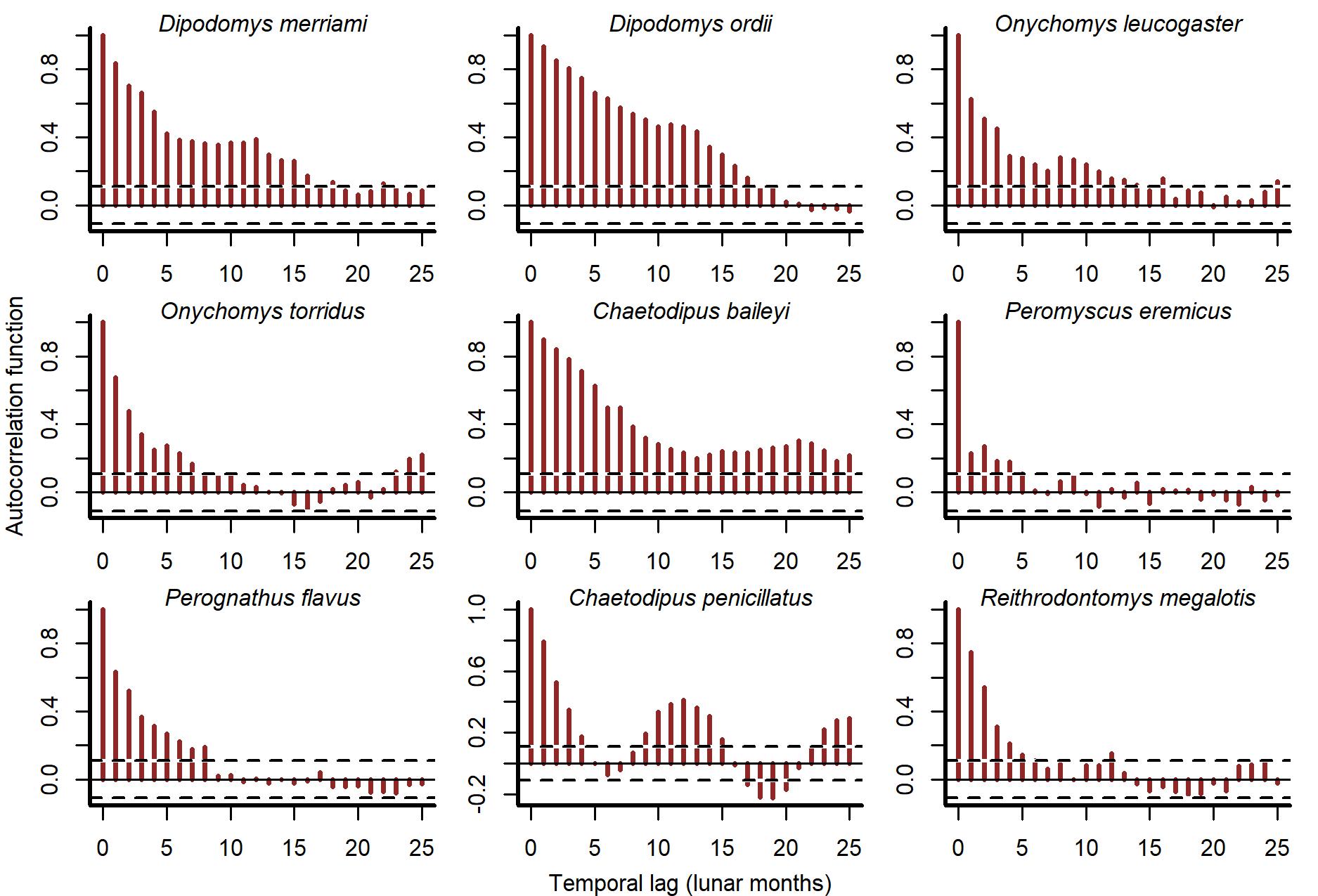

Supplement: Supplemental Information 2 — Dashed lines show values beyond which the autocorrelations are considered significantly different from zero. [file peerj-13-18929-s002.jpg]

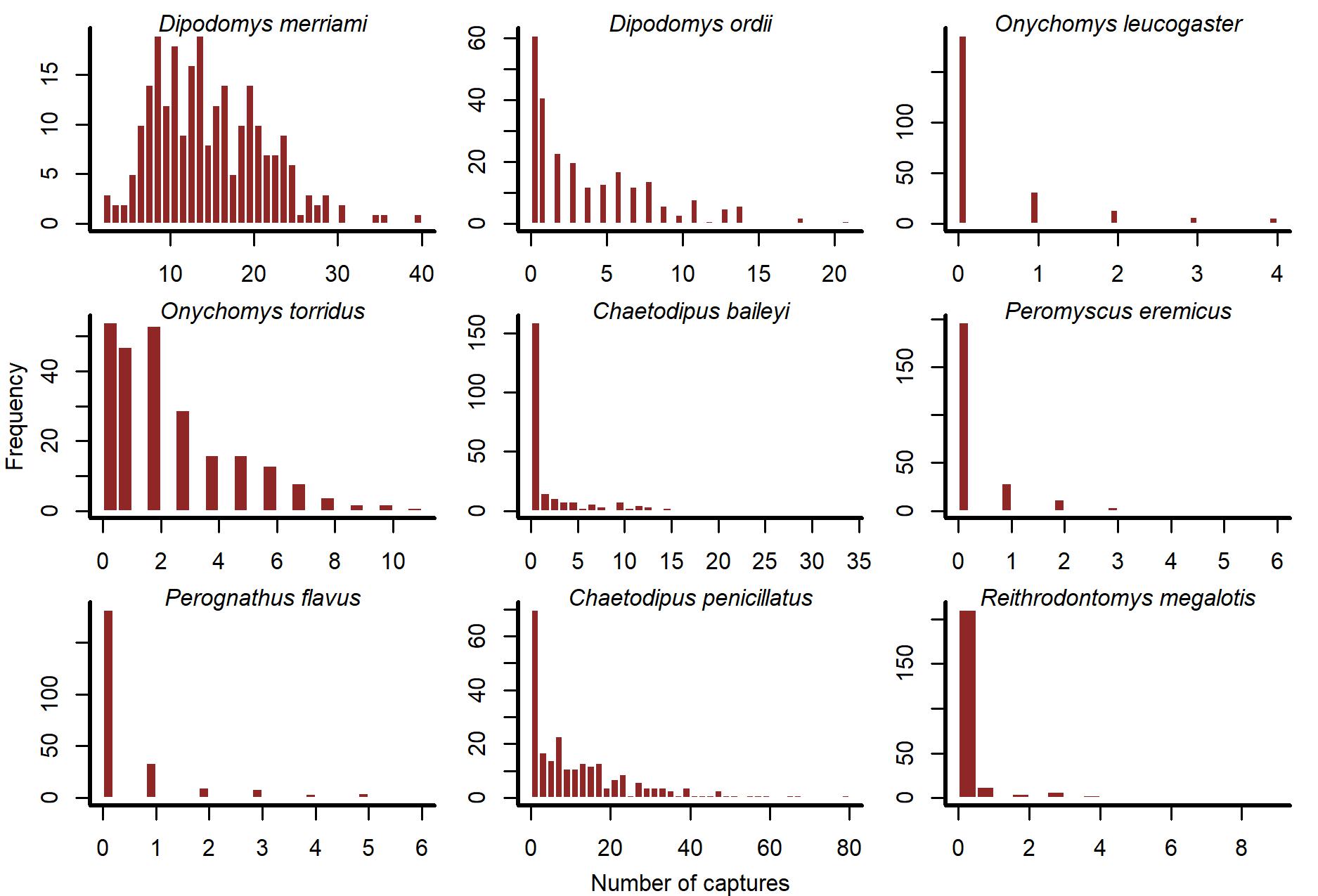

Supplement: Supplemental Information 3 — Counts represent total captures across long-term control plots, sampled at each cycle of the lunar moon. [file peerj-13-18929-s003.jpg]

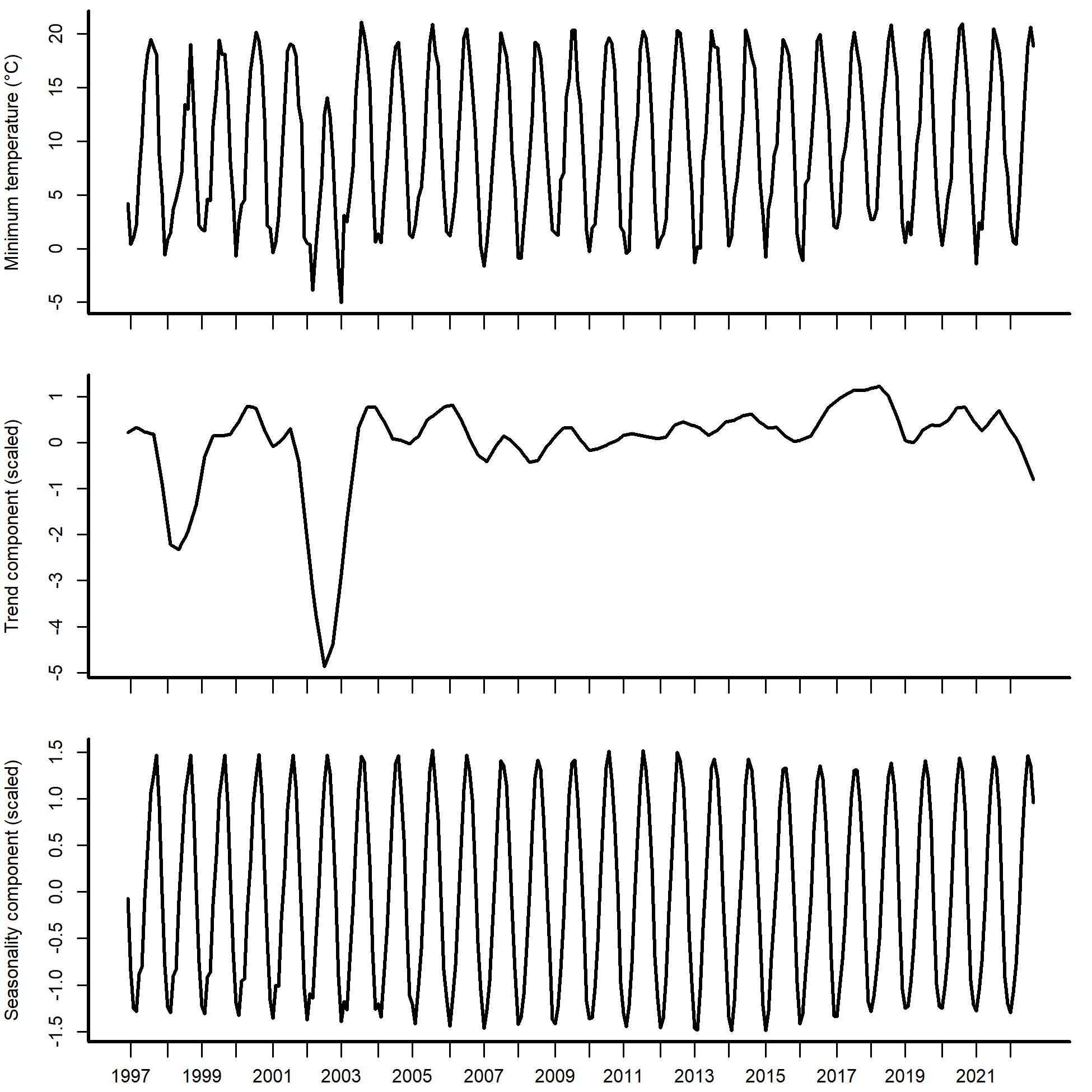

Supplement: Supplemental Information 4 — The top panel shows the raw time series. The middle plot shows the estimated long-term trend (calculated using a Loess regression to the de-seasoned time series). The bottom plot shows the time-varying estimate of seasonality (calculated using a Loess regression that smooths across years). [file peerj-13-18929-s004.jpeg]

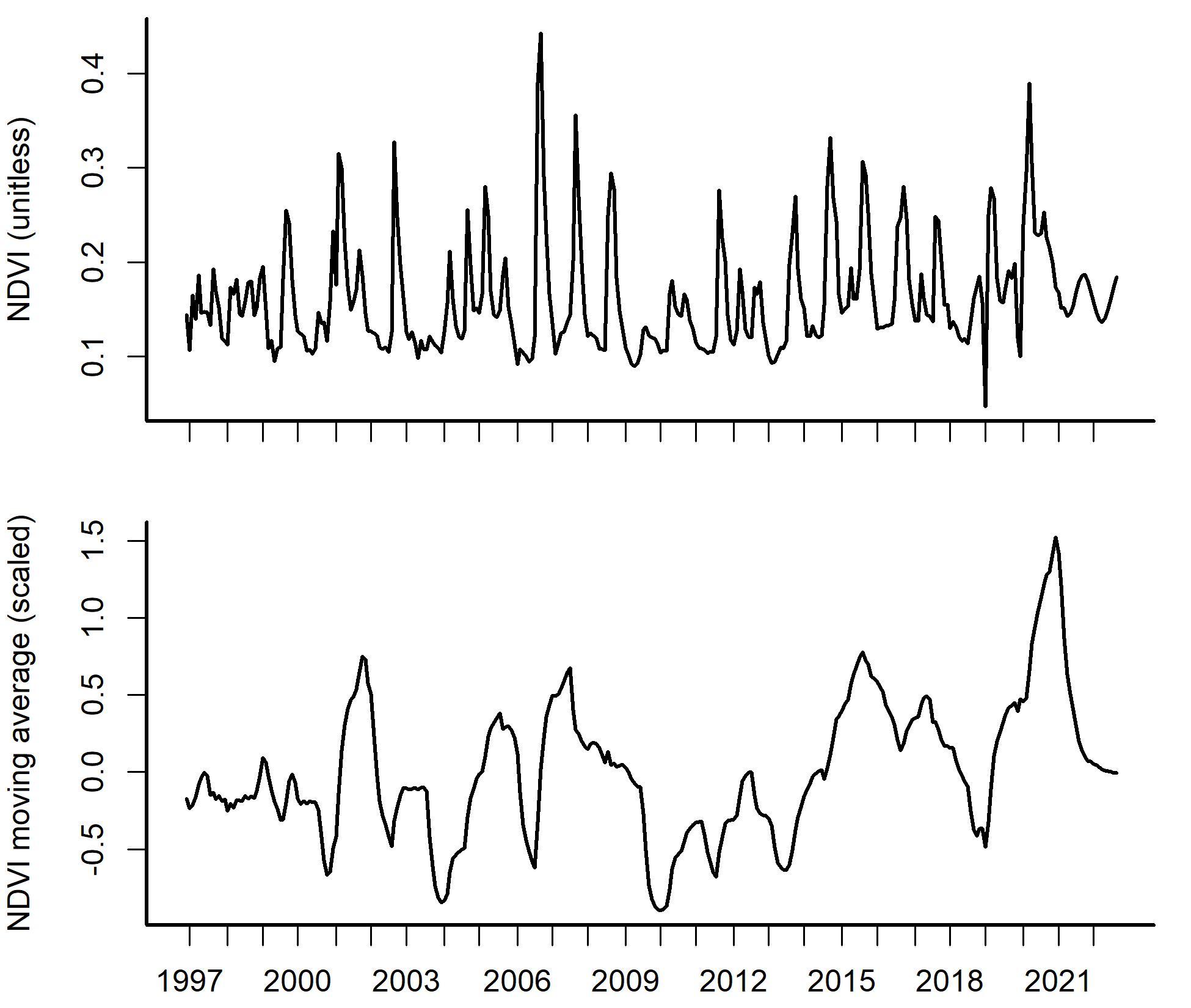

Supplement: Supplemental Information 5 — Top panel: observed Normalized Difference Vegetation Index (NDVI) time series for the period December 1996 – August 2022, with obvious seasonal fluctuations. Bottom panel: a 12-month moving average that represents smooth, gradual changes in NDVI at the study site. [file peerj-13-18929-s005.jpeg]

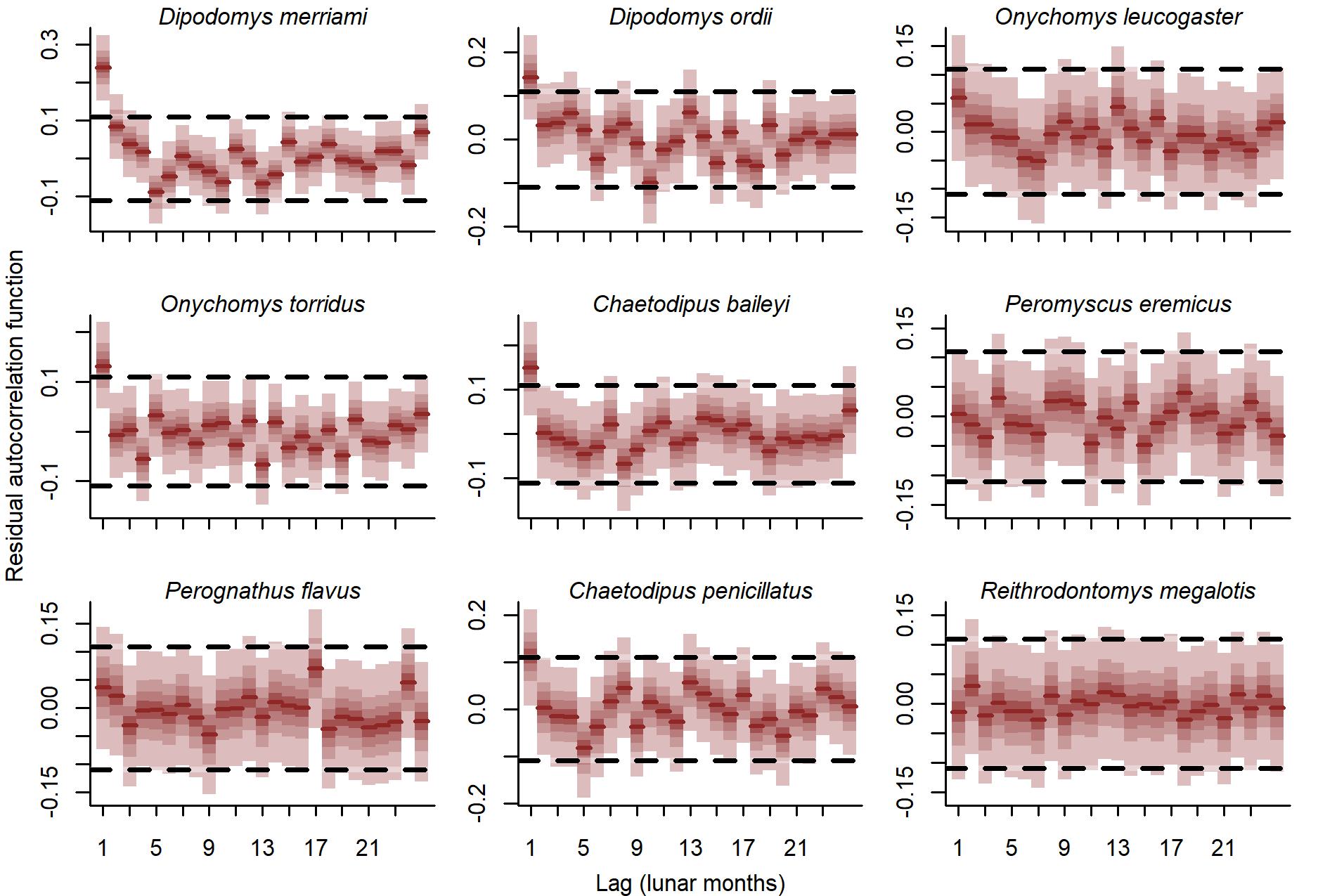

Supplement: Supplemental Information 6 — Ribbon shading shows posterior empirical quantiles (90th, 60th, 40th and 20th). Dark red lines show posterior medians. Dashed lines show values beyond which the autocorrelations would be considered significantly different from zero in a Frequentist paradigm. [file peerj-13-18929-s006.jpeg]

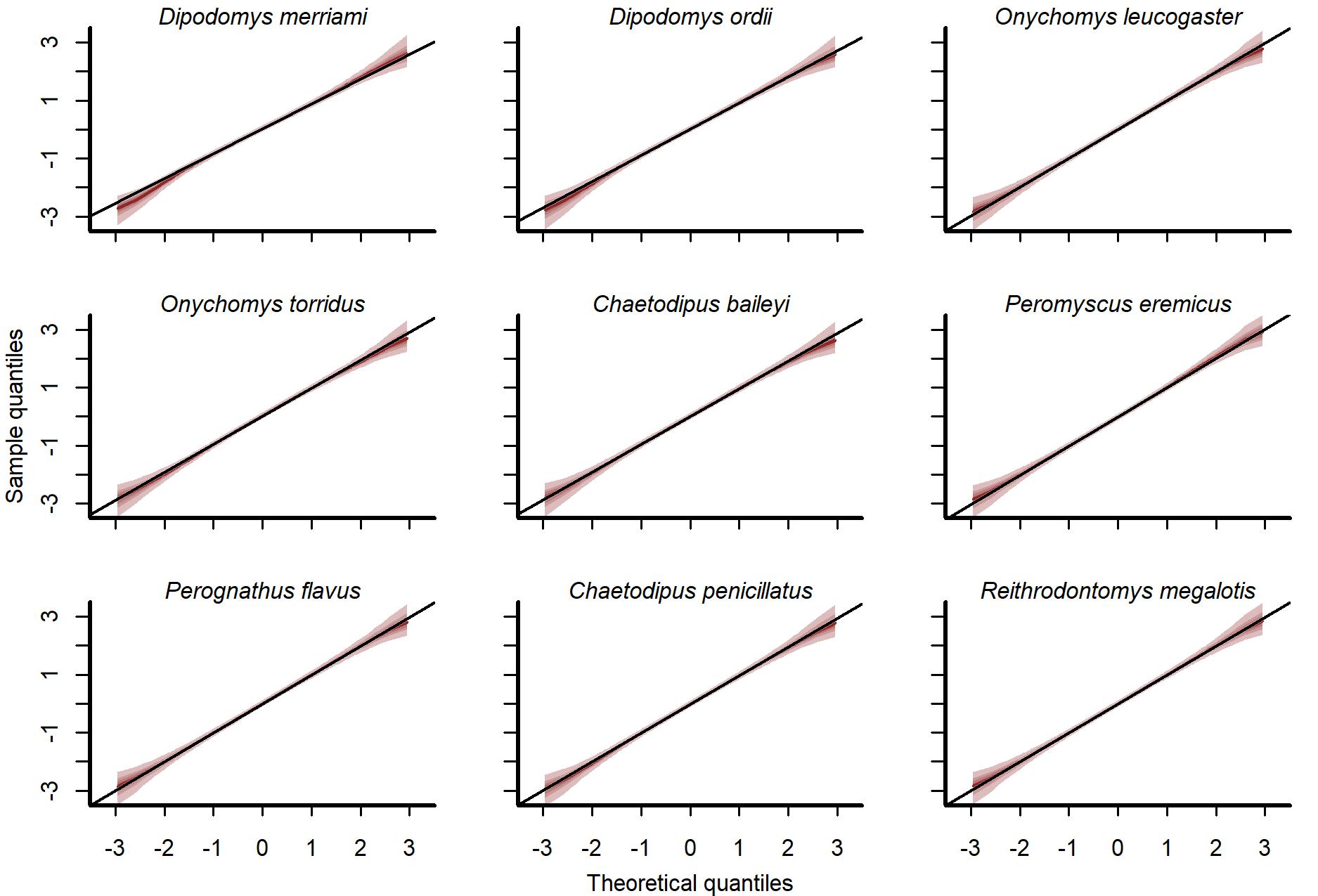

Supplement: Supplemental Information 7 — Ribbon shading shows posterior empirical quantiles (90th, 60th, 40th and 20th). Dark lines show posterior medians. [file peerj-13-18929-s007.jpeg]

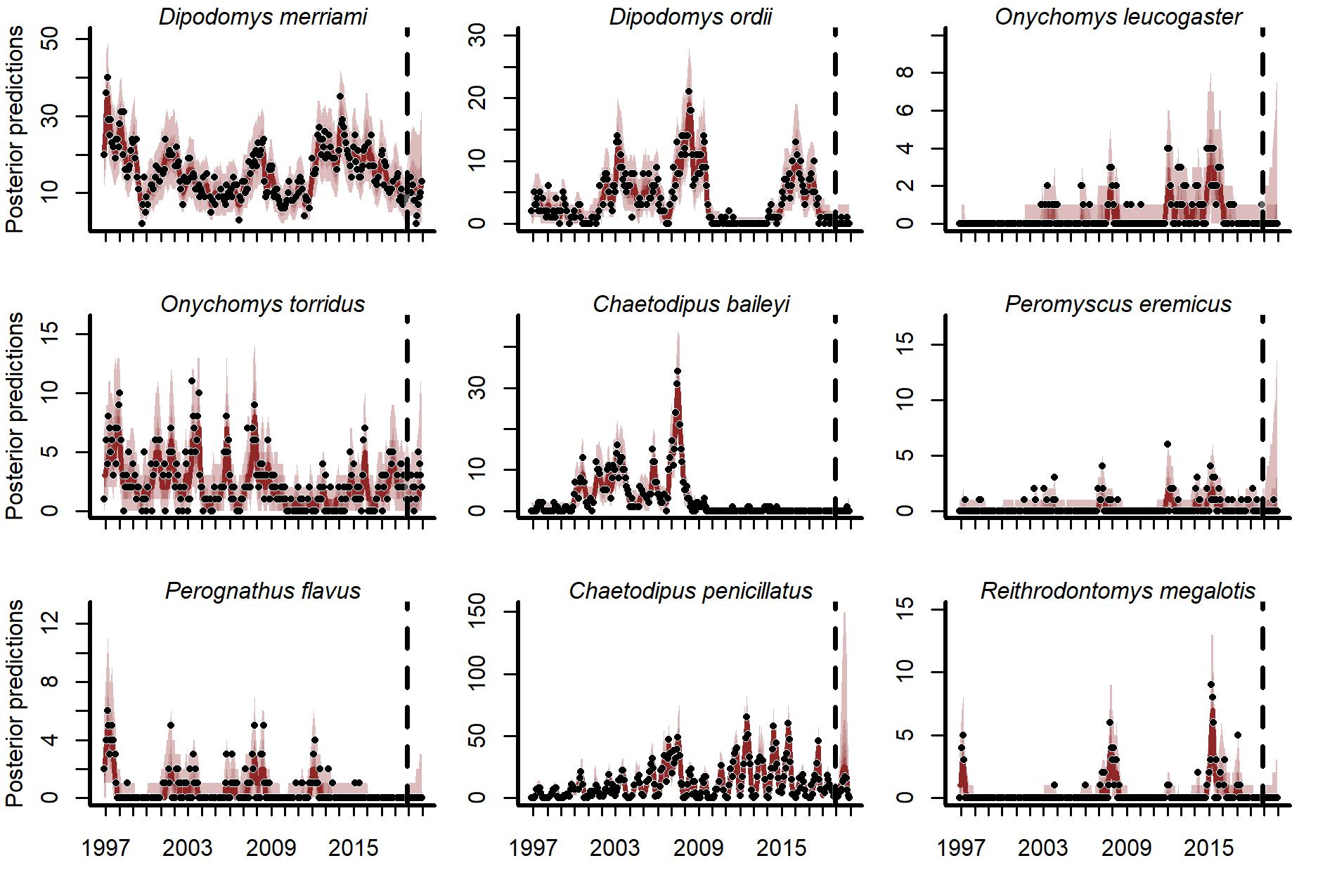

Supplement: Supplemental Information 8 — Latent state estimates were scaled to unit variance for comparisons. Ribbon shading shows posterior empirical quantiles (90th, 60th, 40th and 20th). Dark lines show posterior medians. Points show observations. [file peerj-13-18929-s008.jpeg]

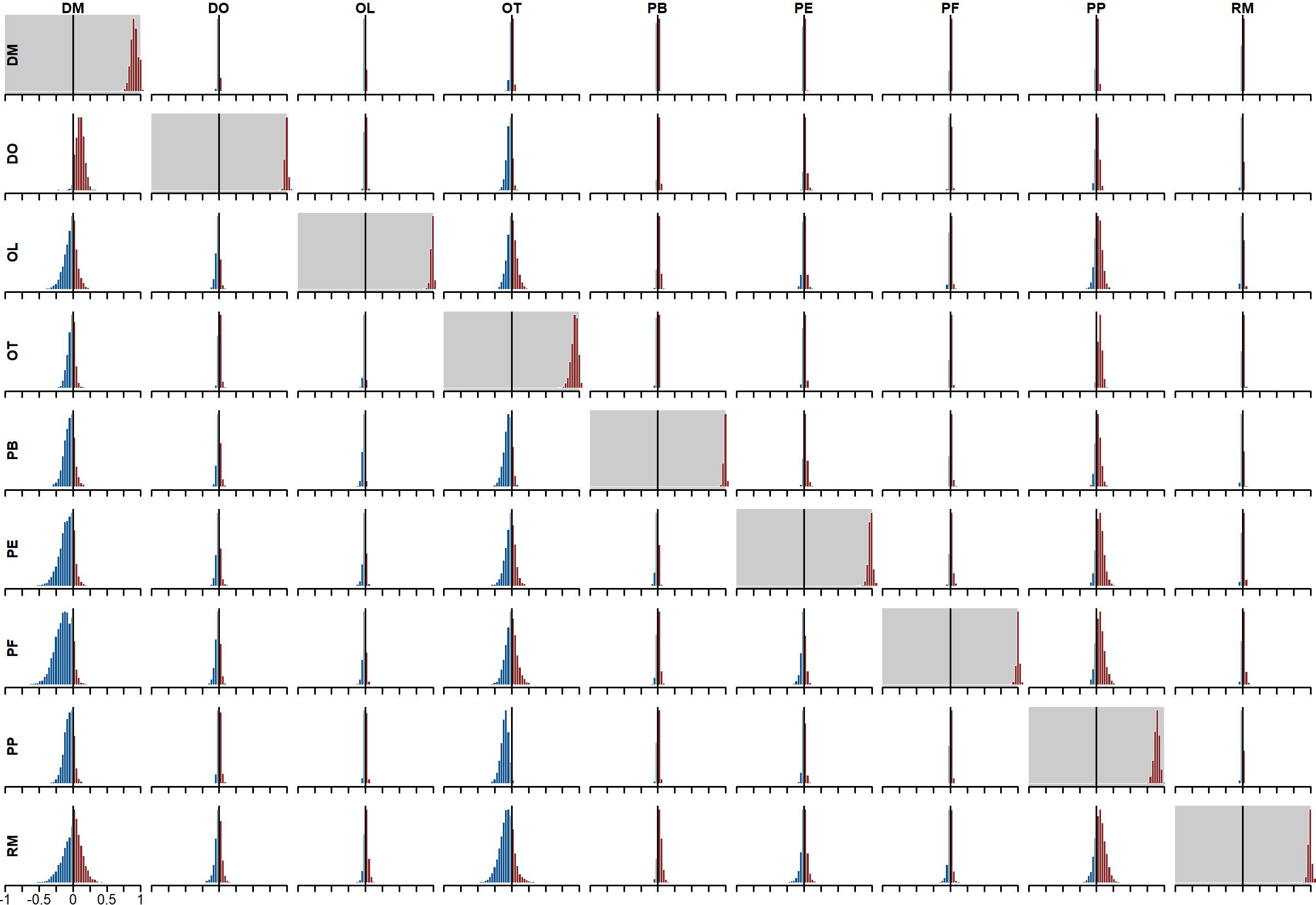

Supplement: Supplemental Information 9 — Off-diagonals represent cross-dependencies. For example, the entry in captures the effect of DO’s state at time t - 1 on the current state estimate for DM (at time t). Diagonals (with grey shading) represent autoregressive coefficients (the effect of a species’ state at time t on its own state at time t - 1). Colours indicate the proportion of probability mass at or below zero (in blue) vs above zero (in red). DO, Dipodomys merriami; DO, Dipodomys ordii; OL, Onychomys leucogaster; OT, Onychomys torridus; PB, Chaetodipus baileyi; PE, Peromyscus eremicus; PF, Perognathus flavus; PP, Chaetodipus penicillatus; RM, Reithrodontomys megalotis. [file peerj-13-18929-s009.jpeg]

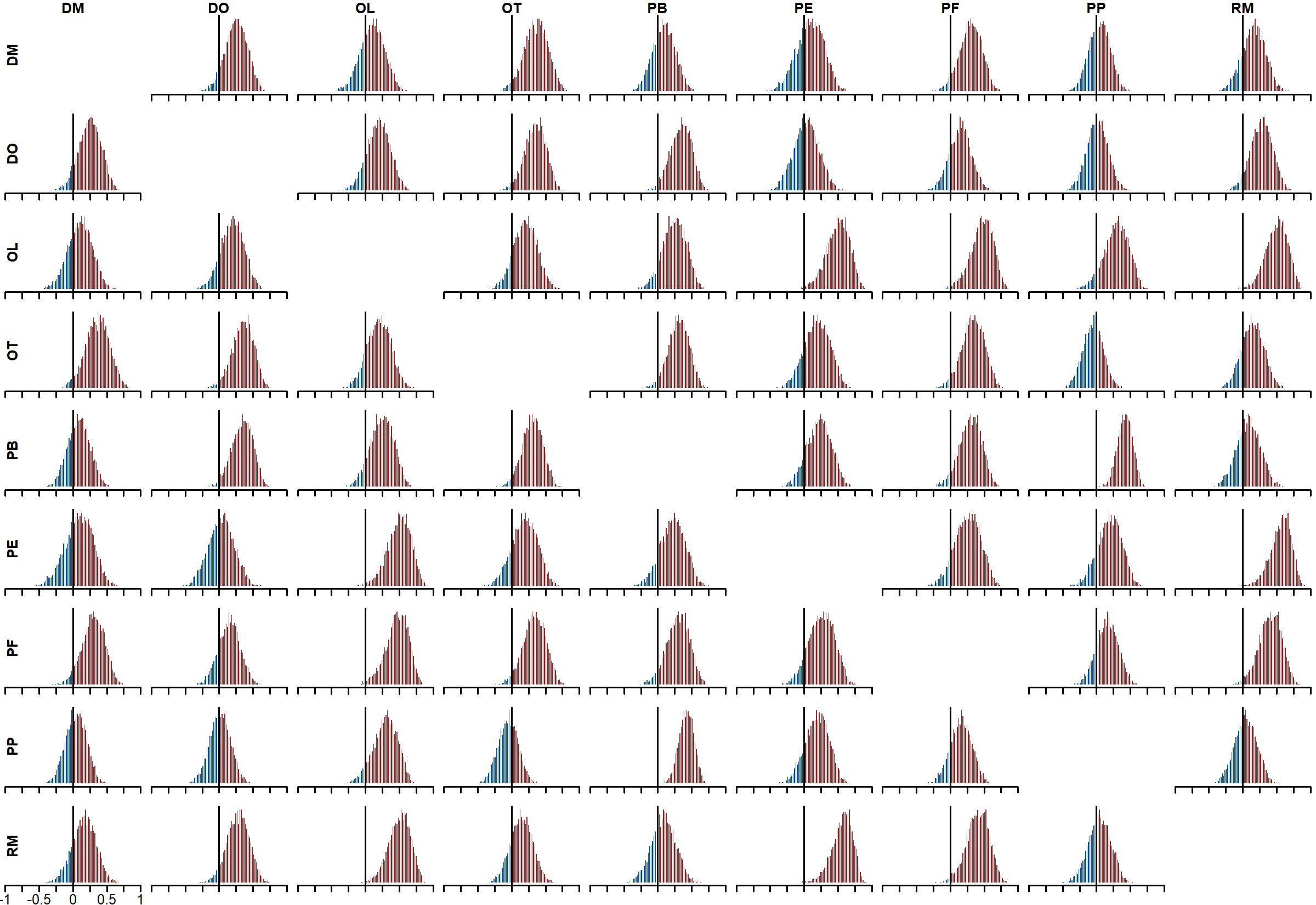

Supplement: Supplemental Information 10 — Colours indicate the proportion of probability mass at or below zero (in blue) vs above zero (in red). DO, Dipodomys merriami; DO, Dipodomys ordii; OL, Onychomys leucogaster; OT, Onychomys torridus; PB, Chaetodipus baileyi; PE, Peromyscus eremicus; PF, Perognathus flavus; PP, Chaetodipus penicillatus; RM, Reithrodontomys megalotis. [file peerj-13-18929-s010.jpeg]

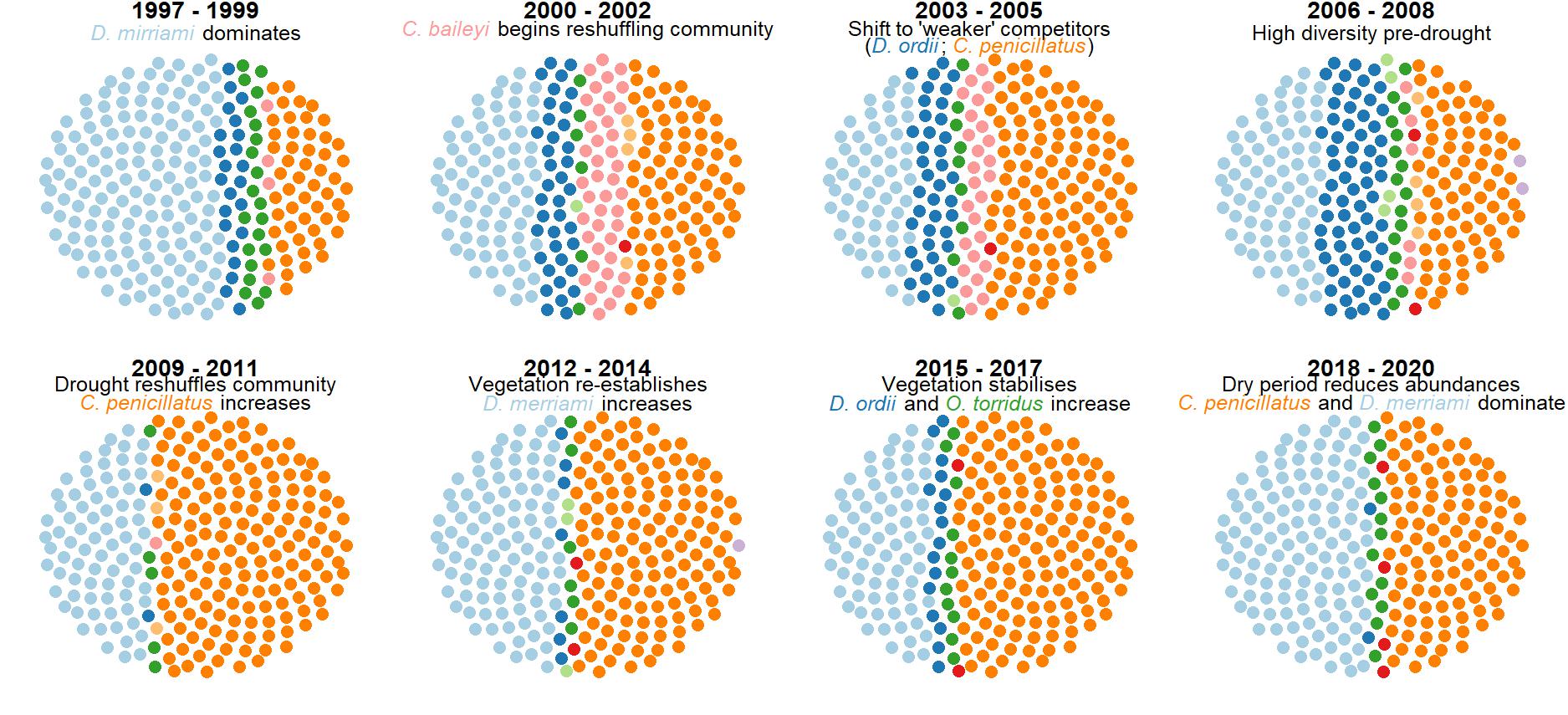

Supplement: Supplemental Information 11 — Using the GAM-VAR model’s posterior predictive distribution, we simulated communities of 200 individuals at different timepoints to investigate how well the model captured known community transitions. Colours represent different species. [file peerj-13-18929-s011.jpg]

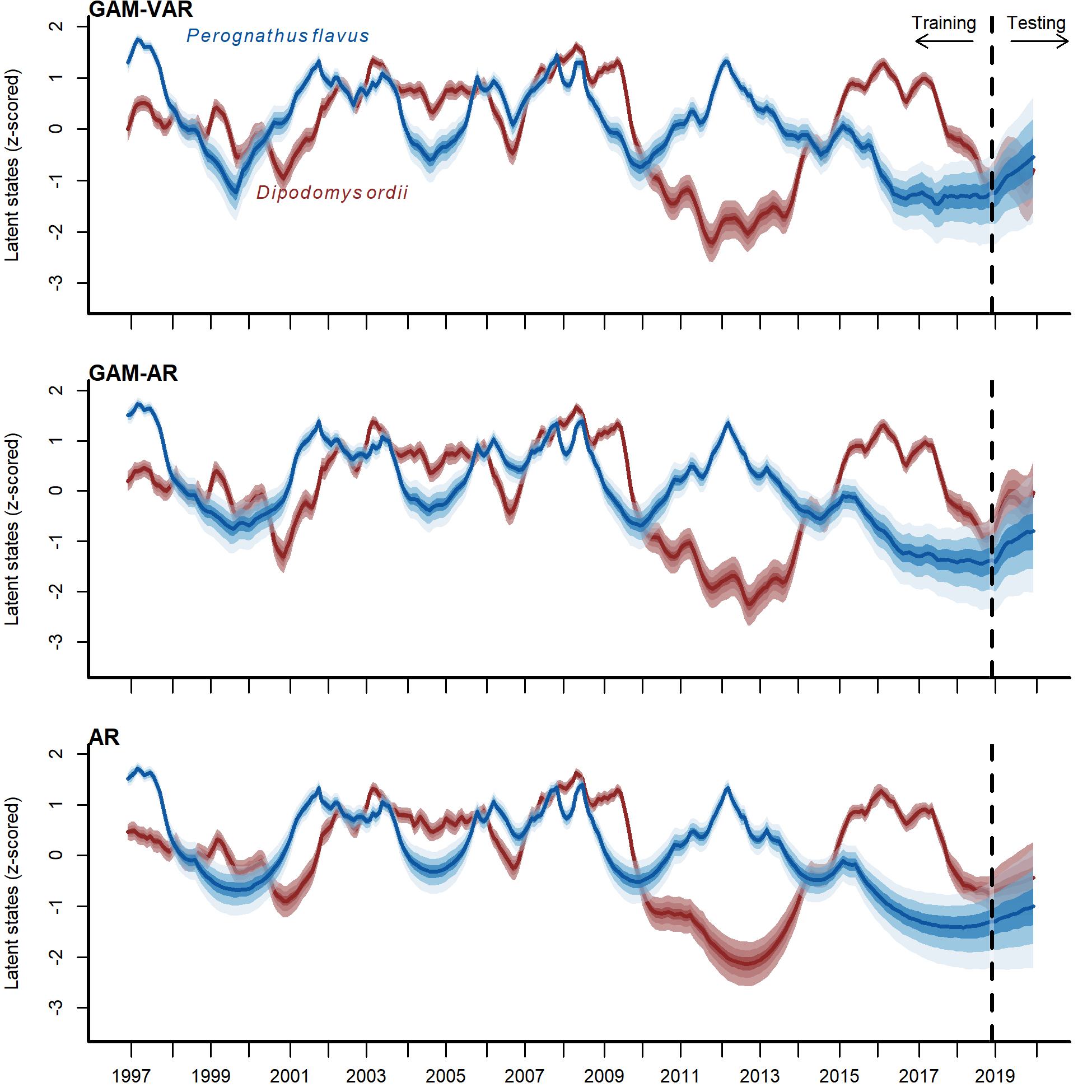

Supplement: Supplemental Information 12 — Trends were scaled to unit variance for comparisons. Ribbon shading shows posterior empirical quantiles (90th, 60th, 40th and 20th). Dark lines show posterior medians. [file peerj-13-18929-s012.jpeg]

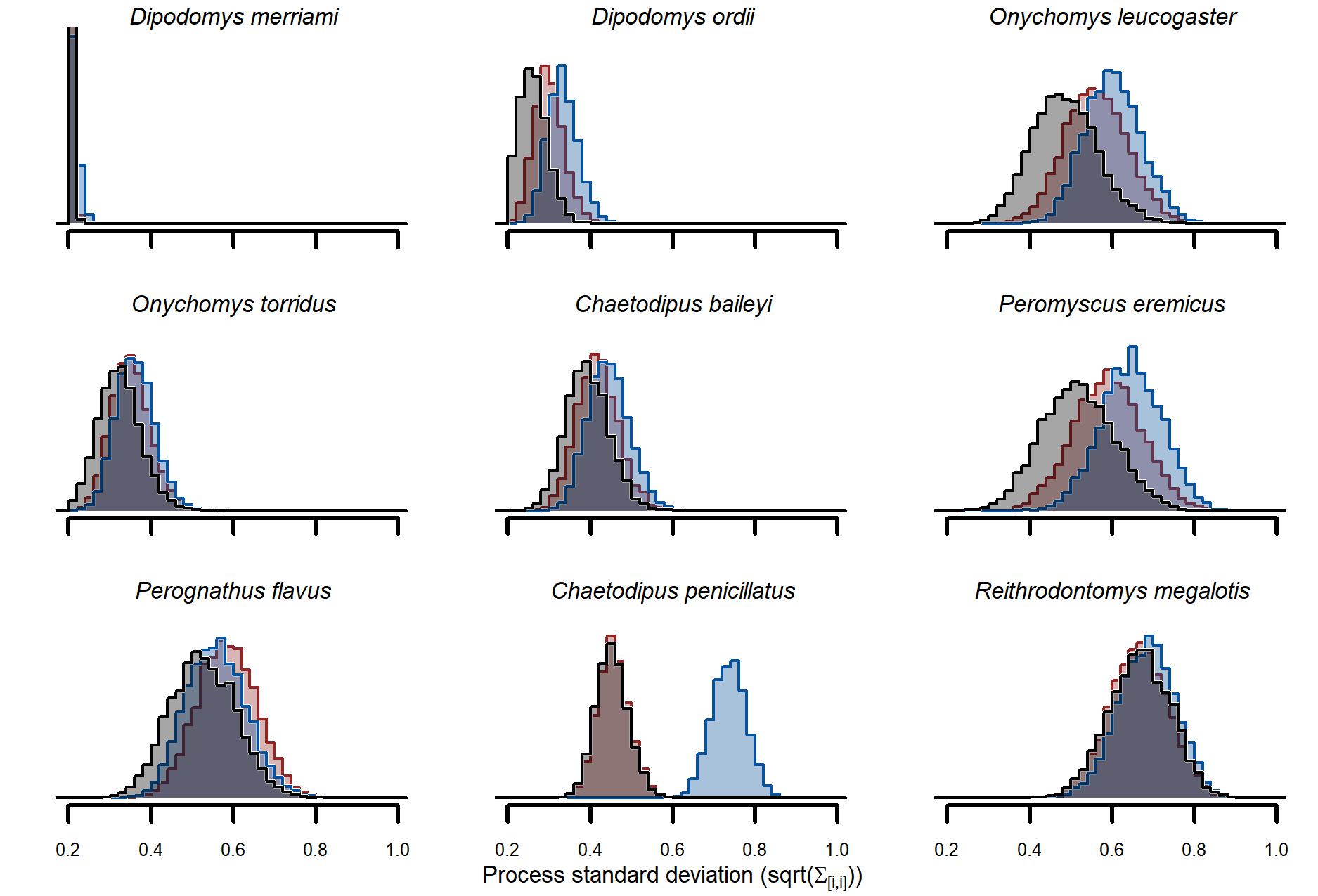

Supplement: Supplemental Information 13 — Estimates are the square root of diagonal parameters from the trend covariance matrix (Sigmavar) for the GAM-VAR (black), GAM-AR (red) and AR (blue). [file peerj-13-18929-s013.jpeg]

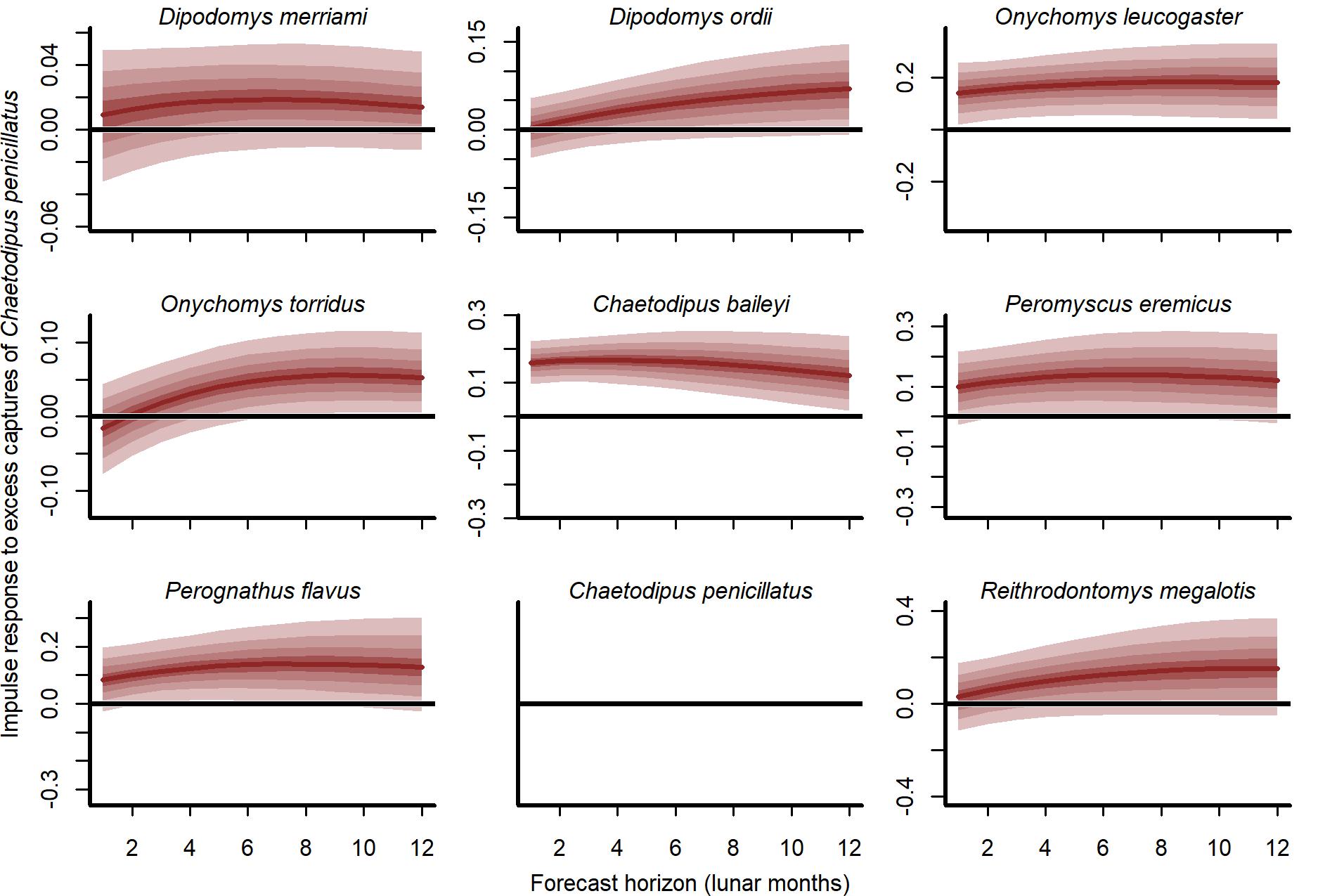

Supplement: Supplemental Information 14 — Ribbon plots show how mean captures (μ, on the log scale) are expected to change over the next six months if three additional C. penicillatus individuals are captured. Ribbon shading shows posterior empirical quantiles (90th, 60th, 40th and 20th). Dark red lines show posterior medians. [file peerj-13-18929-s014.jpg]

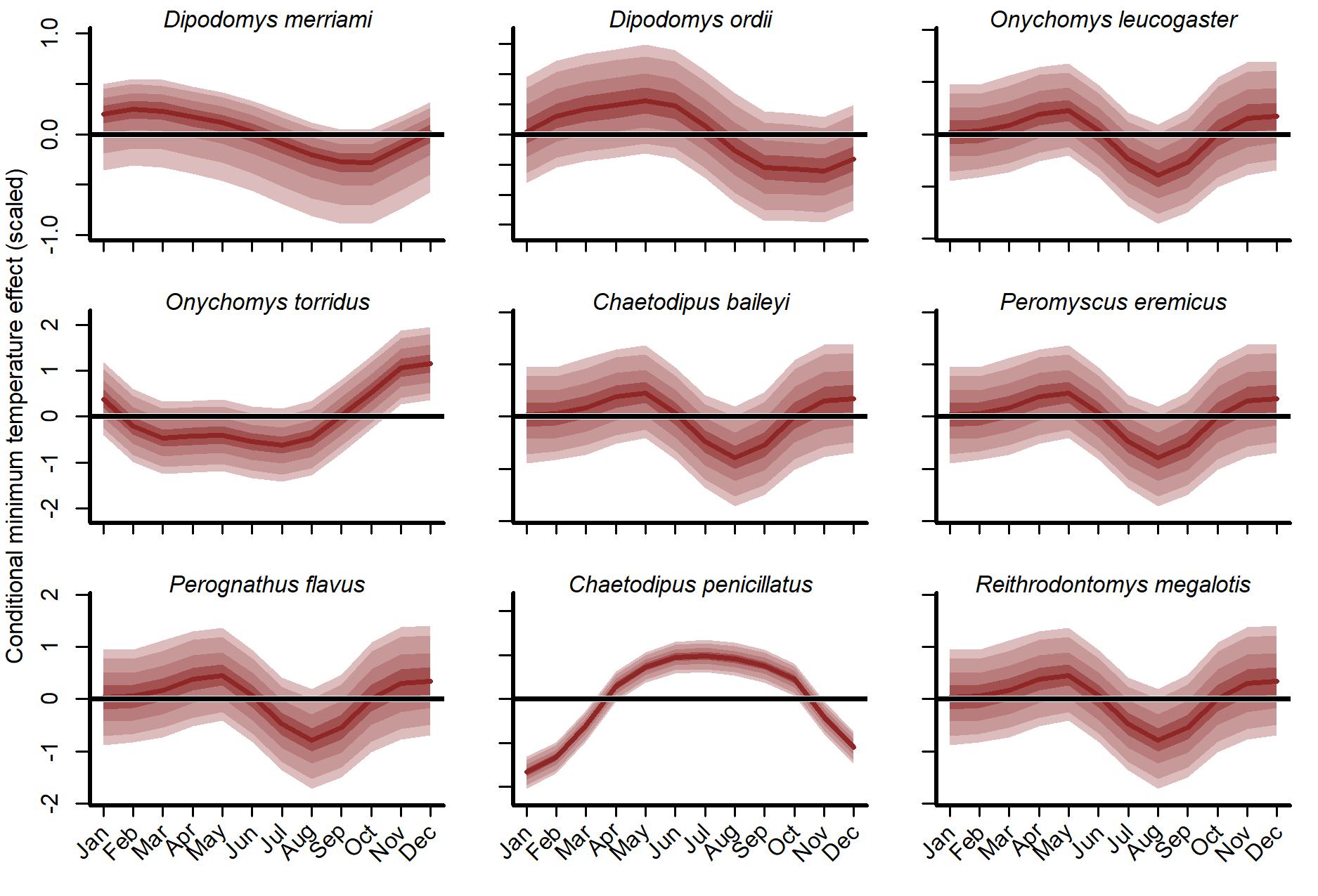

Supplement: Supplemental Information 15 — All other effects were ignored. Functions for O. leucogaster, C. baileyi, P. eremicus, P. flavus and R. megalotis were drawn solely from the global function. Functions for other species were the sum of the global function and a species-specific deviation function. Estimates were scaled to unit variance for comparisons. Ribbons show posterior empirical quantiles (90th, 60th, 40th and 20th). Dark red lines show posterior medians. [file peerj-13-18929-s015.jpeg]

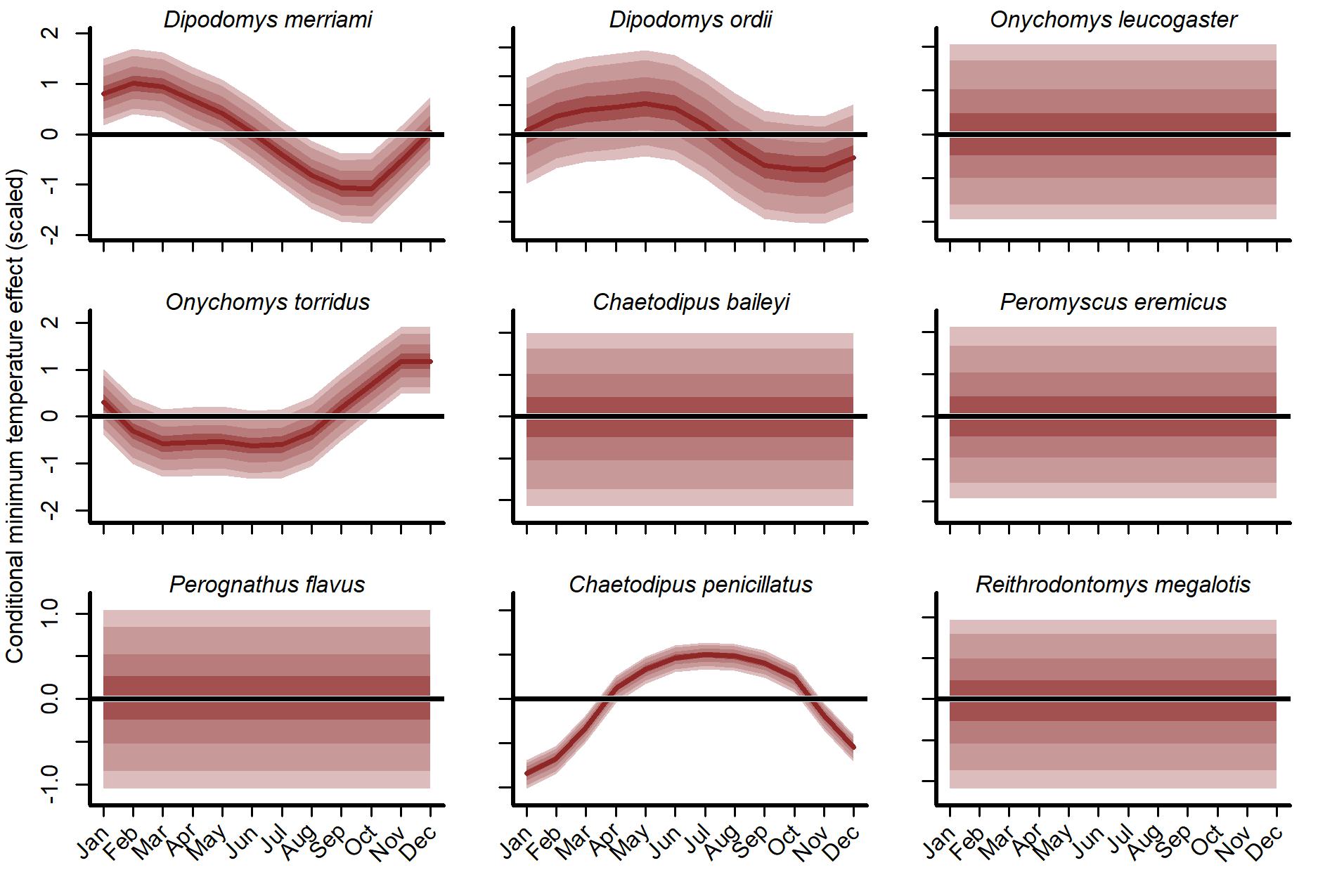

Supplement: Supplemental Information 16 — All other effects were ignored. Functions for O. leucogaster, C. baileyi, P. eremicus, P. flavus and R. megalotis were drawn solely from the global function. Functions for other species were the sum of the global function and a species-specific deviation function. Estimates were scaled to unit variance for comparisons. Ribbons show posterior empirical quantiles (90th, 60th, 40th and 20th). Dark red lines show posterior medians. [file peerj-13-18929-s016.jpeg]
